# Ohnologs and SSD Paralogs Differ in Genomic and Expression Features Related to Dosage Constraints

**DOI:** 10.1093/gbe/evad174

**Published:** 2023-09-30

**Authors:** Zoe Vance, Aoife McLysaght

**Affiliations:** Smurfit Institute of Genetics, Trinity College Dublin, Dublin, Ireland; Smurfit Institute of Genetics, Trinity College Dublin, Dublin, Ireland

**Keywords:** gene duplication, whole-genome duplication, duplicability

## Abstract

Gene duplication is recognized as a critical process in genome evolution; however, many questions about this process remain unanswered. Although gene duplicability has been observed to differ by duplication mechanism and evolutionary rate, there is so far no broad characterization of its determinants. Many features correlate with this difference in duplicability; however, our ability to exploit these observations to advance our understanding of the role of duplication in evolution is hampered by limitations within existing work. In particular, the existence of methodological differences across studies impedes meaningful comparison. Here, we use consistent definitions of duplicability in the human lineage to explore these associations, allow resolution of the impact of confounding factors, and define the overall relevance of individual features. Using a classifier approach and controlling for the confounding effect of duplicate longevity, we find a subset of gene features important in differentiating genes duplicable by small-scale duplication from those duplicable by whole-genome duplication, revealing critical roles for gene dosage and expression costs in duplicability. We further delve into patterns of functional enrichment and find a lack of constraint on duplicate retention in any context for genes duplicable by small-scale duplication.

**Significance**

Duplicate genes created by whole-genome duplication have a different evolutionary trajectory than those created by small-scale processes, with the two sets of genes differing in various important biological properties. However, understanding this phenomenon has been hampered by study differences. This study provides a comprehensive and rigorous comparison of a broad range of genetic features and points to gene dosage effects as the major differentiator.

## Introduction

Gene duplication has long been considered an important mechanism in shaping the gene content and structure of genomes including those of vertebrates (Ohno 1970; Dehal and Boore 2005; Nakatani et al. 2007; Putnam et al. 2008; Simakov et al. 2020; Nakatani 2021). It plays a major role in the creation of new genetic content to be acted on by evolutionary processes and has been frequently implicated in innovations and novel adaptations. Gene duplication can be classified by scale into whole-genome duplication (WGD) and small-scale duplication (SSD). WGD events are implicated in the emergence of novel functional innovations and species radiations, presumably through creation of broad adaptive potential (Jaillon et al. 2004; Scannell et al. 2006; van Hoek and Hogeweg 2009; Qi et al. 2021). By contrast, SSD involves the duplication of much smaller regions of DNA and therefore a smaller quantity of genes. Nonetheless, SSD paralogs are often implicated in adaptation to specific niches (Chen et al. 1997; Desjardins et al. 2012; Kondrashov 2012; Hughes et al. 2018).

The duplicability of a given gene, that is the likelihood of its evolutionary duplication and long-term retention, differs depending on the mechanism of duplication, with retained WGD duplicates rarely successfully duplicated by SSD (Maere et al. 2005; Makino and McLysaght 2010). This striking pattern implies that there is much to be learned regarding the mechanisms of duplicate retention in comparing the two groups. Can we characterize the type of gene likely to successfully duplicate by each mechanism and make inferences about the evolutionary processes at play?

One clear candidate to explain these differences is dosage balance constraint (Papp et al. 2003; Birchler and Veitia 2012). Under this model, duplicate copies of dosage-sensitive genes come under pressure for retention post-WGD to preserve dosage balance but cannot be duplicated outside of a WGD context for the same reason. Paralogs retained after WGD (ohnologs) are known to be enriched for functions associated with dosage sensitivity (Blomme et al. 2006; Brunet et al. 2006). Their evolutionary patterns of gene duplication and copy number variation support the inference of dosage constraints (Makino and McLysaght 2010; Rice and McLysaght 2017b; Defoort et al. 2019), but there are still large gaps in our understanding of duplicability differences. Naturally, many genes do not fall exclusively into one category or the other, that is, some genes retained following WGD also experience SSD. Nonetheless, comparison of these sets of genes should yield insights into the nature of the constraints impacting duplicate retention. A broad range of features have been compared in the existing literature (He and Zhang 2005; Casneuf et al. 2006; Chapman et al. 2006; He and Zhang 2006; Guan et al. 2007; Hakes et al. 2007; Amoutzias et al. 2010; Jiang et al. 2013; Zhu et al. 2013; Keller and Yi 2014; Banerjee et al. 2017; Qiao 2018; Defoort et al. 2019; Qi et al. 2021; Brasó-Vives et al. 2022), with a general pattern emerging that ohnologs are large, complex genes with highly constrained sequence evolution, whereas SSDs are shorter and less constrained. Singletons, which are not observed as possessing retained duplicates, lie intermediate in most cases. However, existing studies cover a wide breadth of lineages and duplicate definitions, precluding meaningful comparison.

Here, we perform a comprehensive comparison of duplicate groups in the human genome, allowing us to combine a broad spectrum of genomic, proteomic, and expression traits under a consistent set of definitions and with the possibility to identify confounding factors. We find that dosage-related features and the number of unique protein domain types present are the strongest distinguishing features between SSDs and ancestral vertebrate (2R) ohnologs. Patterns of functional enrichment additionally inform our overall picture of duplicability, indicating that while 2R ohnologs are generally refractory to duplication by SSD there is no particular constraint preventing retention of SSD-duplicable genes in duplicate following WGD. Combining these findings gives us a view of duplicability wherein gene dosage is the primary driving factor of duplicate retention post-WGD and gene families that include paralogs originating from both mechanisms may most usefully be considered as SSD-duplicable genes with chance ohnolog retention. This view of gene duplicability is consistent with previous reports spanning many diverse taxa, consolidating and uniting them under a single study design with internally consistent analysis protocols.

## Results

### Duplication Mechanism Correlates with Gene Length and Composition Features

We classified 19,548 human genes as one of singleton (no duplicate copies detected within vertebrates), SSD paralog, or ohnolog. For stringency, we assessed paralog pairs using three ohnolog data sets and made the final assignment based on the majority classification for a given gene across three published data sets (table 1; see Materials and Methods). Across all data sets singletons make up the largest class, with SSD and WGD genes more evenly split.

**Table 1:** Duplicate Classification by Data Set

Category	Nakatani et al.	Makino and McLysaght	Singh et al.
WGD pairs	13,934	7,074	6,799
Non-WGD pairs	124,274	131,134	131,409
Excluded pairs:			
Retroduplicated pairs	22,065	23,177	23,135
Prevertebrata duplications	83,668	86,692	87,010
Presumed SSD pairs	18,541	21,265	21,264
WGD genes	3,111	5,814	5,738
SSD genes	3,844	3,180	3,134
Singletons	10,398	8,538	8,697
Excluded genes	2,195	2,016	1,979
Final gene counts:			
WGD	5,327		
SSD	3,352		
Singleton	8,932		

Various sequence composition and gene structure-based features have been studied previously in relation to duplicate category differences, mainly in the context of yeasts and plants. Most agree that WGD genes are longer than other groups in terms of total genomic length (Jiang et al. 2013; Zhu et al. 2013) and that WGD genes possess more structural domains (He and Zhang 2005; Jiang et al. 2013). For other features, the trend is less clear with some reports claiming protein coding sequence length is greater in WGD genes (He and Zhang 2005; Jiang et al. 2013) but others finding no difference (Qiao 2018). Similarly, contradictory results were observed for %GC3 content (G+C content at third codon positions) in two different plant genomes (Jiang et al. 2013; Qiao 2018). Other features have not been examined multiple times and so consistency cannot be judged, for example intrinsic disorder (highest in WGD genes, lowest in SSDs, Banerjee et al. 2017) and average intron length (higher in genes retained post-WGD, Jiang et al. 2013).

**Fig. 1.**
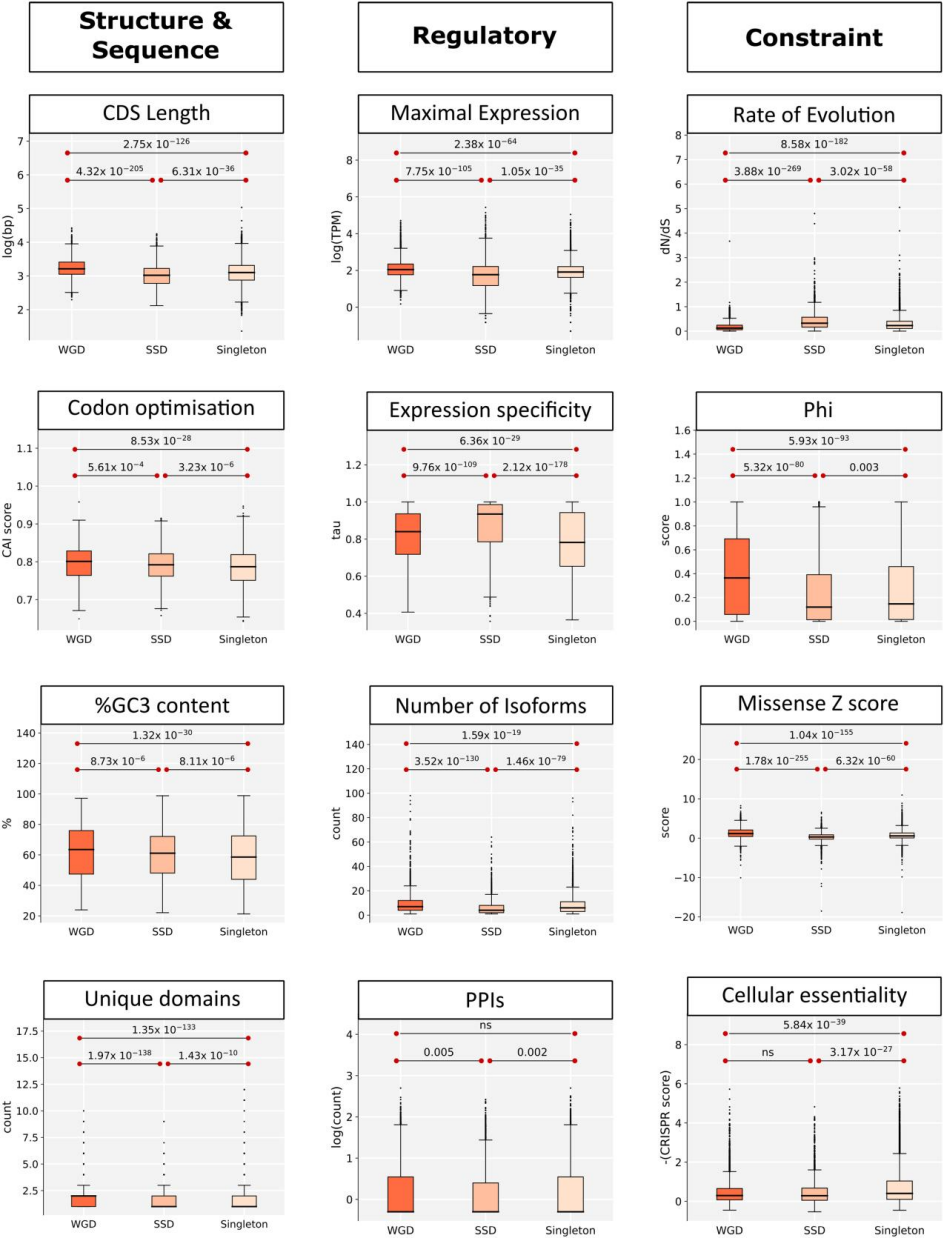
Comparisons for representative features. For each feature, different categories of gene were compared using the Mann–Whitney *U* test. All *P*-values are Bonferroni corrected. Rate of evolution given by dN/dS, missense Z score by the *Z*-score of missense variation in a gene compared to a null model (higher Z score indicating higher intolerance of missense mutation), Phi is the probability of haploinsufficiency.

Our analysis confirms the longer length of WGD genes. Notably, length-related features such as genomic length, CDS length, and intron count are among the most dramatically different between WGD and SSD genes, along with the number of domains/unique domains, followed by further intron-related features such as % intron coverage (fig. 1; supplementary fig. 1, Supplementary Material online). Sequence composition features such as %GC3, codon usage and intrinsic disorder also differ between the duplicate types but much more weakly; both sets of duplicable genes seem to differ more from singletons than from each other in these cases.

Assuming a causal link, there are multiple possible explanations for the difference in lengths and duplicability. One possibility is that the mechanics of tandem gene duplication by nonallelic homologous recombination may bias the size of gene likely to undergo duplication by this method, as larger genes with more functional elements may be less likely to successfully duplicate in their entirety. However, the existence of large segmental duplications that have occurred throughout primate evolution (Bailey and Eichler 2006) suggests that this should not be a significant factor. The longer length of WGD genes is consistent with a “zero-sum game” model of duplicability (Rice and McLysaght 2017a) which implicates the resource depletion costs of duplicating longer genes as a limiting factor in contexts such as SSD where the overall capacity for expression has not increased in tandem with the gene copy number. However, the actual selective impact of such expression costs may not be sufficient to produce these observations in organisms such as human with low $N_{e}$ (Wagner 2005). Thus, the reasons behind these differences remain uncertain.

### WGD Paralogs Have More Complex Regulation Than Other Genes

Previous work on features which impact the regulation of gene expression and interactions gives a clear picture that WGD genes occupy a considerably more complex regulatory context than other groups (Casneuf et al. 2006; Defoort et al. 2019). Within our analysis of features falling into this grouping, the most striking difference is that seen for expression specificity, with SSD genes showing much more narrow expression than either of the other two groups, followed by WGD genes being more specifically expressed than singletons (fig. 1, supplementary fig. 2, Supplementary Material online). Isoform count and maximal expression level also show robust differences across all comparisons, with WGD genes showing highest expression and number of isoforms and SSD genes lowest for both features. Differences in the number of regulatory motifs and protein–protein interactions (PPIs) are comparably small, though WGD genes possess significantly more regulatory motifs than the other two groups and SSDs possess fewer PPIs. The weak difference in PPI count is surprising given dosage balance would predict ohnologs to be enriched for protein complex members, but we cannot rule out that it is merely an artifact of the data set, perhaps due to the absence of indirect interactions within, say, a protein complex, or the absence of interactions reliant on post-translational modifications which are known to be enriched in ohnologs (Amoutzias et al. 2010).

As SSD genes show a strong bias towards narrow expression, we chose to investigate expression level on a per-tissue basis to determine if it is generally true that WGD genes are more highly expressed than SSDs or if SSD genes are the higher expressed group in the tissues where their expression is concentrated. Even when only genes expressed in a given tissue (expressed at 1 transcript per million [TPM] or higher) are considered, the median expression of WGD genes is always higher than SSD genes, indicating WGD genes are the more highly expressed group regardless of tissue context (supplementary fig. 3, Supplementary Material online).

### WGD Genes are More Constrained Than Other Genes

Sequence conservation and constraint may be the most studied category of features with respect to duplication type differences. Evolutionary rates of paralogs are often presumed to reflect relaxation of constraint owing to redundancy, but there is evidence that SSD duplicability is higher for less constrained, faster evolving genes (O’Toole et al. 2018; Vance et al. 2022). In the case of WGD, ohnologs appear to be more constrained: WGD genes evolve more slowly than others (Brunet et al. 2006; Qiao 2018); are more likely to have synonymous substitutions than nonsynonymous (Chapman et al. 2006); and are more often essential than SSDs (Makino et al. 2009).

We examined evolutionary constraint in the duplicate groups by comparing evolutionary rate and population genetics-based metrics (missense Z score, pLI, Phi, ${\text{S}}_{het}$, RVIS, EvoTol, and LoFtool—see Materials and Methods), as well as direct estimates of essentiality in cell lines (Wang et al. 2015). Across comparisons, evolutionary rate, missense Z score, and RVIS are among the most distinct between duplication categories (fig. 1; supplementary fig. 4, Supplementary Material online). These two population-based measures represent two of the three measures which include missense mutations in the estimation rather than only nonsense, frameshift, or splicing mutations, and thus are consistent with the observations regarding evolutionary rates ($d_{N}/d_{S}$, which we interpret as a measure of sequence constraint—see Materials and Methods). This suggests that these groups differ in constraint not only in terms of avoiding total loss of function but also in sequence constraint more generally. The largest differences for other metrics are seen when comparing WGD genes to either SSD genes or singletons, but not when comparing SSD genes to singletons. For example, this pattern is clear for both pLI and Phi, metrics which are used in defining haploinsufficiency. This suggests a difference in dosage constraint between WGD genes and other groups but not between the groups not retained post-WGD.

### Major Differences between Duplicability Groups are Largely Explained by Duplicate Longevity

**Fig. 2.**
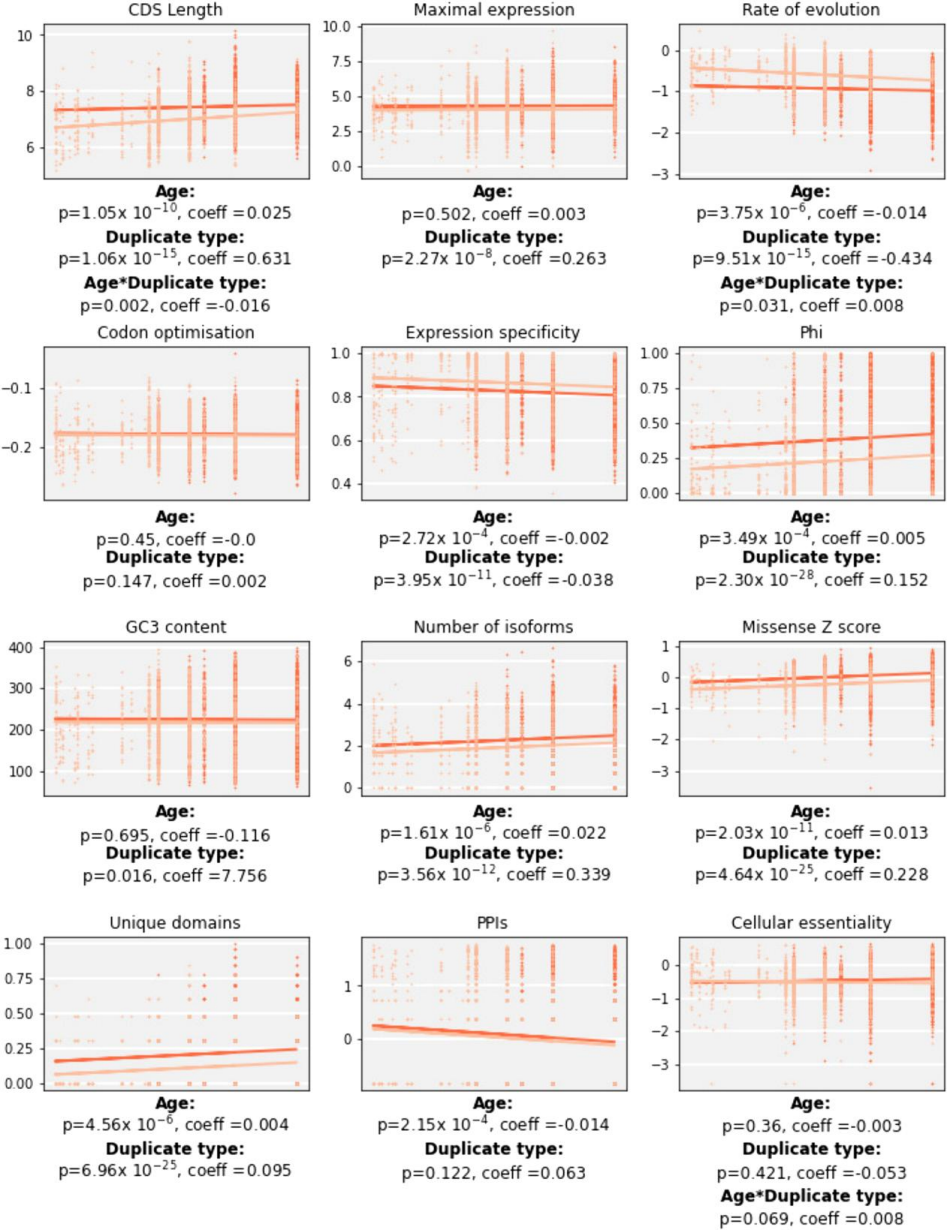
Representative features regressed on duplicate age and duplicate type. Scatter and regression line for each feature with age increasing left to right. WGD shown in darker color, SSD in lighter. Coefficients and associated *P* values for each predictor are given, including for the interaction term where applicable (see Materials and Methods). Units for the features are as in previous figures but transformed according to formulas given in supplementary table 1, Supplementary Material online.

SSD and WGD genes do not differ only in the mechanism of duplication but also potentially in the timing. The WGD-created duplicates considered here all date to early in the vertebrate lineage (the 2R genome duplications), while SSD duplications may have occurred anywhere in the human lineage from the vertebrate ancestor to the human-specific branch. Identified ohnologs are, by definition, long-lived while only a small portion of SSDs are retained in the long term (Lynch and Conery 2003). Previous work has shown distinct patterns of essentiality and functional constraint acting on long-lived versus short-lived paralogs (Woods et al. 2013). We therefore tested the effect of duplicate “age” on the various features. Here we calculated the oldest age of a duplication event for each vertebrate gene family, and used that as the “age” for every family member (see Materials and Methods for further discussion). We used multiple regression models with duplication age and duplication type as predictors for each feature (fig. 2, supplementary fig. 5, Supplementary Material online). Theoretically, if we still see a significant effect for duplication type in a model controlling for age than we can assume the difference between mechanisms exists independently of age.

For the most part, features where duplication type has no significant effect when controlling for age either did not significantly differ in our earlier pairwise comparisons or differed only weakly relative to other features (codon adaptation, %GC, PPIs, cellular essentiality, EvoTol; supplementary fig. 5, Supplementary Material online). For the remaining features where age has an effect within SSDs (genomic length, CDS length, mean intron length, evolution rate, and pLI), we examined whether older SSDs approach the same values as WGDs. (See Materials and Methods and supplementary table 1, Supplementary Material online for features including a significant interaction and how these were determined.) For all these features except for pLI, the SSD and WGD values converge with increasing age. Nonetheless, WGDs remain slightly longer and slower evolving than SSDs of the same age (except in the case of mean intron length; supplementary fig. 5, Supplementary Material online).

### Duplicability Groups are Defined by a Small Subset of Features

**Fig. 3.**
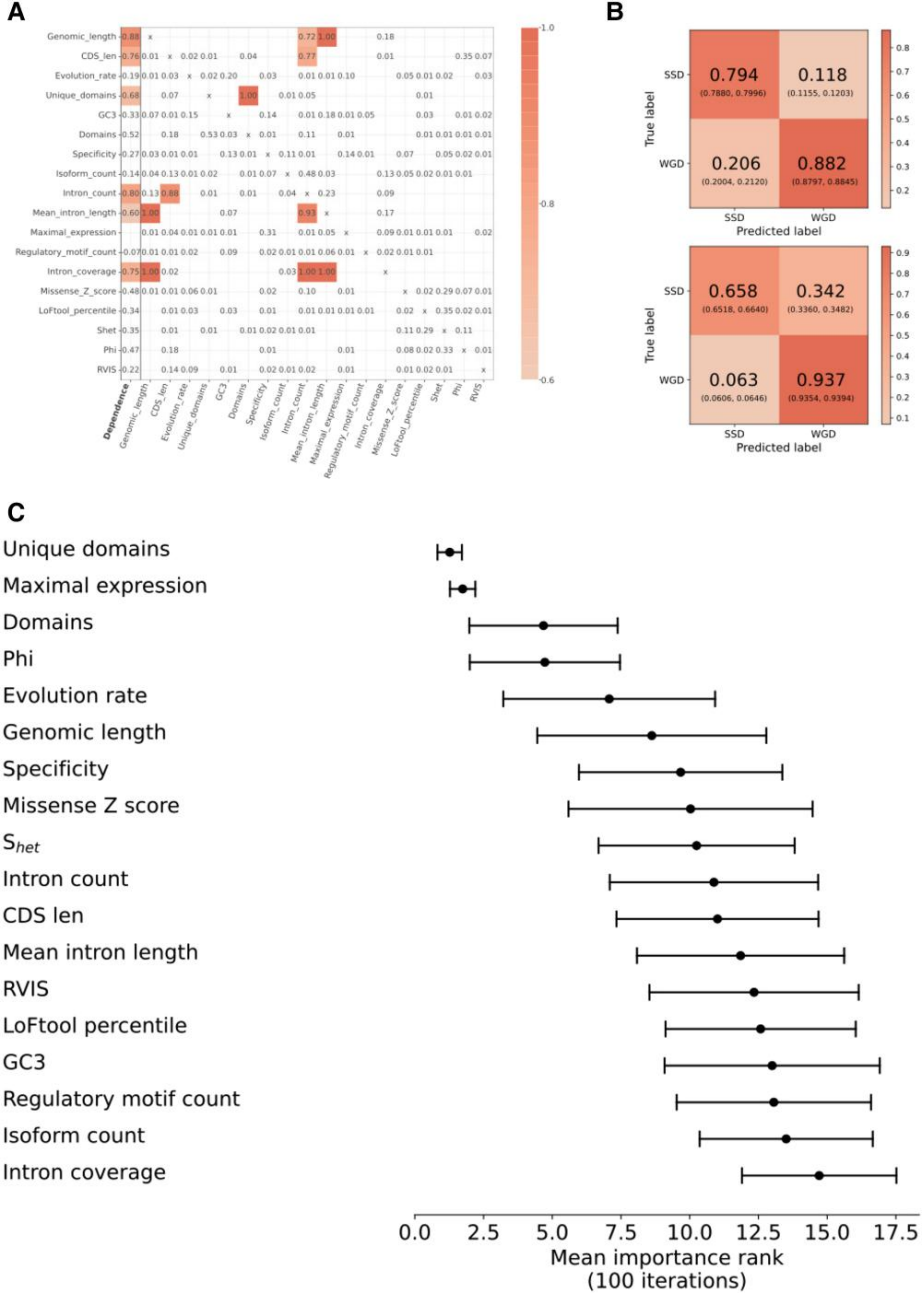
Duplicate type prediction controlling for age differences. (*A*) Feature dependencies, based on a fitted classifier with each feature as the target and all other features as predictors. The “Dependence” column gives the $R^{2}$ value for the model that is how well the feature can be predicted by the others. Other values give the importance of each variable in the given predictive model. Values are bounded at 0 and 1, zero values not shown. (*B*) Normalized confusion matrix for the random forest classifier, values normalized to 1 within each column (top, showing precision) or row (bottom, showing recall). (*C*) Average rank of each feature within the final feature set of 18 determined by feature importance estimate, averaged across 100 iterations of fitting the classifier, features ordered top to bottom by highest to lowest mean importance. Error bars indicate 1 s.d.

Our final aim in relating gene features to duplicability was to integrate the observed feature differences to determine the most important features in distinguishing duplication classes. To this end, we built a random forest classifier to classify the two duplication types using a final set of 18 features (listed in fig. 3*C*) which differed significantly between WGD and SSD genes in both the original pairwise comparisons and after controlling for age, where we used values from the regression analysis for each feature to control for age (fig. 3).

We have used age-controlled data in our final analysis due to the clear influence of age biases in the duplicate classes. The impact of controlling for age is evident in multiple respects. We see an increase in overall model accuracy compared to a model based on raw data (mean accuracy of 0.8625 (95% CI: 0.8604–0.8646) across 100 iterations compared to 0.7963 (95% CI: 0.7938–0.7988)) ; we also see, improvement in a bias towards classifying older duplicates as WGD (improvement in SSD recall and precision; fig. 3, supplementary fig. 6, Supplementary Material online). We suspect that removing the influence of duplicate age has had this effect due to the unadjusted model misclassifying older SSD duplicates as WGD duplicates. As there are no younger WGD duplicates, this effect was one-sided. Age is also a clear correlate of many of the features considered. Any relevance of dependency between features is eliminated on controlling for duplicate age (see Materials and Methods), suggesting much of the shared information between these features is age-related and contributed to misclassification of older SSDs.

Our final model indicates that the most informative features for the classification of duplicate type have links to gene dosage or expression cost. Probability of haploinsufficiency is clearly a dosage requirement-related feature, with the higher probability of haploinsufficiency in ohnologs relating to dosage balance constraints. The higher level of expression in 2R ohnologs has a plausible relationship to expression cost; doubling of expression of highly expressed genes is costly to the cell and may eventually impact overall expression capacity (the “zero-sum game” model of gene expression (Rice and McLysaght 2017b)). Ohnologs should not experience the same issues as the cellular machinery will have been concurrently duplicated, allowing for retention of highly expressed duplicates. A role for the number of unique domains is less clear cut. This feature may reflect the overall complexity of the proteins produced or some degree of higher multifunctionality in ohnologs.

### Functional Profiles of Duplicability Groups Offer Insights into Duplicate Retention Processes

While we find gene dosage to be a key determinant of duplication differences, naturally gene families exist which retain duplicates of both duplication class. Small contributions from other gene features could explain this, but a more complete picture of duplicability requires assessment of gene function. Different classes of duplicate are known to show specific functional enrichments. We replicate these known patterns, finding enrichments for developmental, regulatory and signaling functions in WGD genes as well as depletions for base cellular functions such as translation (supplementary tables 5 and 6, Supplementary Material online; Blomme et al. 2006; Brunet et al. 2006; Hakes et al. 2007; Kassahn et al. 2009; Makino and McLysaght 2010; Session et al. 2016; Qiao 2018; Conant 2020). Similarly, we recover known SSD functional enrichments such as for roles in sensing and immunity (supplementary tables 7 and 8, Supplementary Material online), (Hakes et al. 2007; Qiao 2018), along with the same depletion for base cellular functions seen in 2R ohnologs. The most highly enriched/depleted terms for singletons complement those of the WGD genes with the greatest enrichments in translation and mitochondrial functions (supplementary tables 9 and 10, Supplementary Material online).

**Fig. 4.**
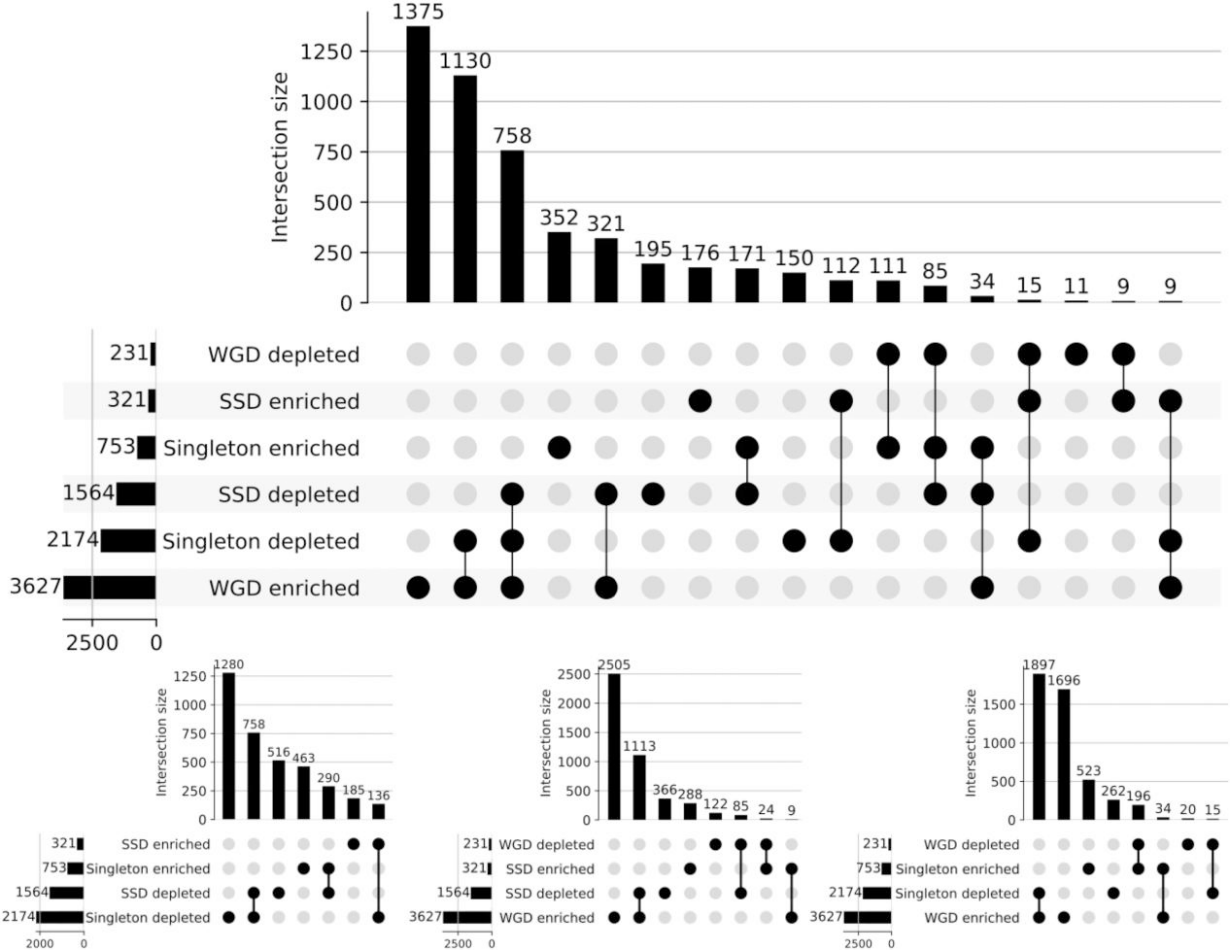
Overlap of enriched/depleted GO terms between categories. Top panel shows intersection size, filled in dots and connections below indicate which groups are overlapping for a given intersection and the side panel shows the total number of GO terms significantly enriched/depleted for each duplicate group. Lower plots are simplified versions of the top plot showing only overlaps between two groups at a time.

Given observed overlaps in the sets of functions most strongly enriched/depleted across groups, we decided to investigate the overall overlaps within all significant enrichments/depletions (fig. 4). We observe a stronger complementary pattern between WGD genes and singletons with 53.3% of terms enriched/depleted in WGD genes showing the opposite trend in singletons, (vs. only 29.5% for SSDs) and 71.4% of terms in singletons showing the opposite pattern in WGD genes (compared to 14.6% for SSDs). On the other hand, singletons and SSDs are the most concordant groups, with 40.2% of SSD terms showing the same enrichment pattern in singletons (compared to 5% for WGDs), though this is driven entirely by sharing of depleted terms.

These shared depleted terms in the two non-WGD groups should represent functions which are preferentially retained post-WGD. That we do not see such agreement between singletons and WGD suggests there is not a similar class of functions promoting SSD retention. This idea is supported by asymmetry between the two duplicate types; 71.2% of SSD depleted terms are enriched in WGD genes, while only 7.5% of terms enriched in SSDs are depleted in WGD genes. Overall, it seems that WGD gene functions are primarily shaped by functions which promote duplicate retention following WGD (purifying selection maintaining the ohnologs) while SSD functions are shaped by avoiding functions of genes which would be detrimental to duplicate in this manner.

## Conclusions

The results presented here reinforce previous studies that have shown that WGD and SSD have significantly different evolutionary outcomes. Numerous previous studies have analyzed the relationship between gene duplication mechanisms and other genetic, genomic, and proteomic features. In this work, we provide a comprehensive analysis of a broad range of characteristics in the context of the vertebrate 2R WGD and vertebrate SSD that benefits from both an internally consistent set of ohnologs, SSD paralogs, and singletons, and, importantly, includes a rigorous examination of potentially confounding features. In particular, the random forest classifier provides insight into which features are making the greatest contribution to the differences in gene duplicability by either mechanism. Comparison of functional enrichments across groups additionally supports the idea that ohnolog retention occurs primarily, though not exclusively, in gene families which are refractory to SSD, while the reverse is not true (there is no evidence to support a large set of genes that are refractory to post-WGD retention).

We examined differences between duplication types across a wide range of features covering gene length, composition, structure, regulation, and constraint. The most extreme differences between SSD and WGD genes generally fall under either features relating to gene length or those relating to constraint on sequence changes. We further showed that these differences cannot be fully explained by differences in duplication antiquity, although gene age does have a dramatic effect on how strongly these features contribute to accuracy in classifying duplicate type. It is significant that these features demonstrate a stronger relationship to duplicate age than duplication mechanism as, within our data set, they are also the features with the most accumulated evidence for differing strongly between the duplicate groups. This clearly shows that duplicate age must be considered when comparing features of duplicate genes.

The features that we find to contribute most strongly to the random forest classifier accuracy on controlling for this age effect indicate a strong influence of gene dosage effects in determining successful duplication, most notably gene expression level and probability of haploinsufficiency. The biological relevance of the dominance of the number of unique domains as a top predictor of duplicate class is less clear within current models of duplicate retention. The fact that the number of unique domains seems to provide information not given by the absolute number of domains (table 2) implicates diversity of function within the gene as perhaps being the relevant parameter. Potentially the presence of multiple types of domain has bearing on gene essentiality, or may impact the probability of retention with a greater number of subfunctions allowing more scope for subfunctionalization (though the latter should apply to both WGD and SSD). Another potential explanation is that the number of unique domains that occur in combination within a single gene is related to the function of these domains. Previous work examining domain combinations (Apic et al. 2001) has found that domains bearing specific functions are more “versatile” in their combinations. Many of these functions (such as regulation and signaling) are enriched in 2R ohnologs, opening the possibility that this feature has captured their functional profile and that perhaps this functional versatility is the underlying feature distinguishing duplicate types.

**Table 2.** Change in Rank for Correlated Features Considered in Isolation for Age-Controlled Model

Feature	Previous Rank	New Rank	Previous Accuracy	New Accuracy
Group 1				
Genomic length	6	6	0.8651	0.8628
CDS length	11	11	0.8651	0.8640
Mean intron length	12	10	0.8651	0.8630
Intron count	10	9	0.8651	0.8664
Intron coverage	18	16	0.8651	0.8624
Group 2				
Unique domains	1	1	0.8651	0.8587
Domains	4	3	0.8651	0.8533

The difference in isoform count was unexpectedly large, with SSD genes having far fewer than either singletons or 2R WGD genes. One potential explanation for this could be the idea of alternatively spliced transcripts acting as “internal paralogs” (Modrek and Lee 2003), with an alternative transcript essentially allowing some novel function to evolve in a similar manner to a copy of the gene. Here, a greater number of isoforms would be viewed as a symptom of copy number constraint, representing one of the few routes to novelty of an otherwise restricted gene. An alternative explanation could be that greater numbers of alternative transcripts somehow impact the likelihood of successful duplication. This distinction is important when considering what role various features may play in duplicate evolution and further work may be required to distinguish between these possibilities for various features, for example as has been done for evolutionary rate differences (O’Toole et al. 2018; Vance et al. 2022).

The overall goal of this work was to confirm and consolidate previous studies of duplicability by creating an integrated view of differences between duplicate categories, so that these differences could be used to make deductions about what promotes or hinders duplicate retention under each mechanism. It is clear from the single feature comparisons that human WGD genes are longer and more broadly expressed as well as being highly constrained and regulated, with the most relevant features implicating dosage and functional diversity as important determinants of duplicate type. It remains to be discovered what about these features drives different evolutionary outcomes following WGD or SSD.

One final outstanding question is whether these findings for vertebrate duplications can be generalized to duplicability in other lineages. While we do not attempt to address this here, and to completely address this question would require further work in defining duplicate status and genetic features in a comparable manner across species, we do note some parallels across lineages. Recent work in amphioxus (Brasó-Vives et al. 2022) indicates significant overlap in genes duplicable within the nonvertebrate chordates and that the functional profiles of duplicated genes are similar to vertebrates. Looking to more diverged lineages, enrichment of immune functions has been observed both in human (this work) and in plants (Qiao 2018), despite huge differences in plant and animal immunity. Commonalities along these lines hint at interesting possibilities for a general framework of ohnolog evolution.

## Materials and Methods

### Data Sources and Processing

#### Paralog and Ohnolog Sets

A list of 19,548 human protein coding genes, as well as a list of human paralog pairs with an estimate of duplication timing (last common ancestor) was obtained from Ensembl v99 (Yates et al. 2019). Regions of macrosynteny originating from the 2R vertebrate genome duplication were obtained from Nakatani (2021) and used to define ohnolog pairs. These pairs were defined as Ensembl paralog pairs that linked macrosyntenic regions and existed in blocks of at least three linking pairs with no more than eight genes separating any two paralogs on the same segment. Additional ohnolog data sets were obtained from Makino and McLysaght (2010) and Singh and Isambert (2019) for cross referencing to generate the final duplicate category assignments.

Considering each ohnolog set separately, a paralogous pair was designated as originating from WGD if the pair was present in the list of ohnolog pairs, and as SSD if not. Pairs designated as SSD were further examined for evidence of retroduplication, as this mode of duplication is outside the scope of this work and it cannot be assumed that these pairs behave similarly to SSDs. Paralog pairs were defined as potentially retroduplicated if one member of the pair has zero introns while the other has three or more, or if at least one member of the pair has zero introns and the other has less than three *and* there is no conserved microsyteny between the duplicates. Microsynteny was defined as having at least one other paralagous pair linking the surrounding region (within five genes either side of the genes being tested), following from methods used in Jun et al. (2009) (supplementary fig. 9, Supplementary Material online). The additional microsynteny check was used for low intron genes as there is a possibility that the second member of the pair is a tandem duplicate and reached zero introns through intron loss rather than retroduplication or, in the case where both members of a pair have zero introns, that the original parent gene had zero introns prior to duplication. The possibility of intron gain in a retroduplicated pair was not considered as intron gain is rare and losses typically outnumber gains (Roy and Penny 2007). Genes were assigned their mode of duplication status based on the pairs they are present in. Ohnologs are present only in WGD pairs, SSD genes only in SSD pairs and genes not present in any paralogous pairs within the vertebrate lineage are designated as singletons. Singletons can be further divided into genes with no paralogs at all and genes with no paralogs since the vertebrate divergence (duplication node as given by Ensembl is Chordata or earlier). Genes belonging to any pair classed as resulting from retroduplication are excluded, as are genes with pairs belonging to more than one duplication type. The final classification for each gene was based on “majority rules” between the three ohnolog data sets.

#### Measures of Gene Essentiality

A measure of cellular essentiality, the CRISPR score, was taken from Wang et al. (2015). This score is derived from a proliferation screen defined as the average ${\text{log}}_{2}$(fold-change) in the abundance of all sgRNAs from the library used which target a given gene, that is the change in sgRNAs causing disruption to a gene. The greater the decrease in the disruptive sgRNAs, the more essential a gene is, so smaller CRISPR scores indicate greater essentiality. The minimum CRISPR score across the four cell lines in the data set (that is the maximum essentiality) was taken. We use the negative of this score and refer to it as “cellular essentiality”.

We also examine a number of population-based measures of gene essentiality. These metrics differ from the CRISPR score as they use modeling to estimate the expected frequency of mutation or loss of function in a given gene and define essentiality based the difference between this expected frequency and that observed. We examine a number of these metrics including pLI (Lek et al. 2016), Phi (Bartha et al. 2015), LoFtool (Fadista et al. 2016), ${\text{S}}_{het}$ (Cassa et al. 2017), RVIS (Petrovski et al. 2013), EvoTol (Rackham et al. 2015), and missense Z score (Samocha et al. 2014).

Of these metrics of essentiality, each has distinct features but there are some commonalities in the statistical methods they employ. RVIS and EvoTol both estimate residual variance after regressing the number of common variants on total number of variants for a given gene, thus defining genes as essential (intolerant of variation) if they have fewer common variants than expected given the total functional variance for the gene. Although they share this framework, these metrics are distinct in the data used with RVIS considering variants using only information from humans, while EvoTol incorporates whether a variant is “damaging” across evolutionary time. Similarly, Phi, pLI, and Shet all use estimates from probabilistic models (Poisson mixture models in the case of Phi and pLI, Bayesian in the case of Shet) to measure tolerance of mutation, estimating probability of haploinsufficiency, probability of LOF intolerance, and the selection coefficient, respectively. The missense Z score quantifies the deviation in missense mutation frequency relative to the expectation of a neutral mutation model, making it the only metric included here which focuses fully on sequence changes rather than including mainly truncating variants. Finally, LoFTool is the only metric included which is built on multiple of the others, combining information from both EvoTol and the missense Z score to consider a breadth of functional information.

#### Expression and Expression Specificity

Gene expression levels (median values, in TPM) were obtained from GTEx (v8) (GTEx Consortium 2020). Expression data for differing developmental stages were obtained through Expression Atlas (fetal expression data from FANTOM5, expression at various stages of prenatal brain development from the Human Developmental Biology Resource and expression at various stages of development, project numbers E-MTAB-3358, E-MTAB-4840, and E-MTAB-6814, respectively). Expression specificity was calculated as the tissue specificity index *τ* which is given by $\tau =\frac{\Sigma_{i=1}^{N}(1-x_{i})}{N-1}$, where *N* is the number of tissues and *x${}_{i}$* is the expression value for the *i*th tissue scaled by the highest expression value for the gene. *τ* essentially represents the average difference across tissues from the maximal gene expression, scaled relative to the maximal gene expression; a value of 0 indicates a housekeeping gene while a value of 1 indicates a tissue specific gene. For the purposes of this calculation, different development stages were treated as separate tissues. This measure of tissue specificity was determined to be the most robust when benchmarked by Kryuchkova-Mostacci and Robinson-Rechavi (2016).

#### Assignment of Duplicate Longevity

We considered the “age” of a given gene to be the age of the oldest duplication node within the vertebrate gene family, with gene family membership defined as all paralogs of a gene originating within the vertebrate lineage, according to duplication timing provided by Ensembl (Yates et al. 2019). For the purposes of comparison we allowed WGD duplicates to take the ages returned by this method, even though the WGD is known to be a single event; this allows for the possible effects of delayed rediploidization (Lien et al. 2016; Robertson et al. 2017; Redmond et al. 2023) but may also reflect phylogenetic inference error. Nonetheless, we prefer to treat the two sets of paralogs the same in this manner to avoid introducing additional comparison artifacts. In order to explore the impact of how long-lived duplicates of a given gene family are likely to be, we assigned a duplicate “age” to individual genes. We chose to use the oldest duplication node present in the family of each gene to capture paralog longevity, though admittedly this is the maximum observed paralog longevity in the family, and not the average nor the most recent duplication for a given gene. Clearly, this reflects something different than the most recent duplicate. However, we were concerned that the age of the most recent duplicate could mask the presence of very long-lived paralogs in a family, and that gene families with a very short paralog half-life would not be distinguished from those with a long half-life. Nonetheless, a potential concern is whether there are large differences between the oldest and most recent duplication date, and what impact this has on our analysis. For ohnologs, this is less of a concern as by definition these genes are long-lived, having arisen in the 2R WGDs. However, for SSDs further attention is warranted. We explored the difference in this metric for SSD genes between using the oldest paralog and using the most recent paralog metric as a given gene’s “age” and find a similar distribution of genes across nodes using either measure (supplementary fig. 10*A*, Supplementary Material online). For many genes, it is the case that there is minimal distance from the oldest to youngest paralogs (supplementary fig. 10*B*, Supplementary Material online). For those where this is not the case, we observe larger gene family sizes (supplementary fig. 11, Supplementary Material online), confirming that this measure accurately captures the long-lived nature of certain duplicates.

#### Other Features

PPIs were obtained from the Human Interactome (Luck et al. 2020). Codon adaptation index (CAI) and proportion of residues in intrinsically disordered regions (IDRs) were determined from CDS sequence using CAIcal (Puigbò et al. 2008) and IUPred (Mészáros et al. 2018), respectively. All other features used were obtained from Ensembl biomart or Ensembl API (or derived from data available through these sources). This included genomic length, CDS length, evolution rate (*dN/dS* with macaque orthologs), number of introns, average intron length, intron coverage, regulatory motifs, protein domains, unique protein domains (from domains), % GC content, and % GC3 content (determined from CDS sequence from Ensembl). In cases where a feature may differ between different transcripts/protein products of a gene, the value for the longest transcript was used.

In the case of “evolutionary rate”, we use the ratio of nonsynonymous mutation rate to synonymous mutation rate ($d_{N}/d_{S}$). Although this value is not a “rate” in strict terms, but rather a ratio of two rates, convention is to refer to it as measuring the rate at which a gene evolves and so we label it “evolutionary rate” here and interpret lower values as “slower” evolution. This metric may also be taken to represent the mode of selective pressure a gene is evolving under, such that values over 1 (excess of nonsynonymous changes) indicate positive selection while values under 1 (depletion of nonsynonymous changes) indicate negative selection. In this context, it can also be interpreted that these groups evolve under differing selective conditions, as shown in previous work examining the selection pressures affecting preservation of the two duplicate types (Ezoe et al. 2021).

### Statistical Methods

Two-tailed Mann–Whitney *U* tests were used for all direct comparisons of features between duplicate types and between duplicates and singletons. *P*-values were Bonferroni corrected for multiple testing when applicable.

Depletion and enrichment of GO terms in each category was determined using gProfiler (Raudvere et al. 2019) using FDR to control for multiple tests with a threshold of 0.05 and using the total gene set as background.

#### Regression Models

Regression models were built to investigate if duplicate differences were explained by differences in longevity of duplicates between duplicate types. Models were built using OLS regression with the statsmodels Python package (Seabold and Perktold 2010), data transformations were selected from none, log transformation, and Box–Cox transformation, according to which minimized deviation from normality (lowest Jarque–Bera test statistic) and whether or not to include an interaction term was determined based on whether adding the term yielded a significant improvement in AIC (a decrease of 2 or more units). Final formulas used for each feature are given in supplementary table 1, Supplementary Material online. Duplicate “age” here is as defined above, with one age unit corresponding to 50MY of divergence time using time estimates from TimeTree (Kumar et al. 2017).

#### Random Forest

A random forest classifier for determining feature importance was constructed with all SSD and WGD genes with values available for all features considered using the scikit-learn Python package (v0.24.1) (Pedregosa 2011). This method was selected over, for example, a regression model as it is a better choice in cases where there may be complex interactions between features.

Selected hyperparameters (i.e., parameters of the model set prior to training—“n_estimators”, “max_features” “max_depth”, “min_samples_split”, “min_samples_leaf”, “bootstrap” were considered) for these classifiers were determined from a randomized grid search with 10-fold cross- validation set to maximize F1 score, with a model trained on the selected gene features. Following this step, the estimated best hyperparameters were the same as defaults except in the case of the number of estimators (39 rather than the default of 100). Classes were weighted ($\text{class}\_\mathrm{weight}=‘‘{\text{balanced}}^{\prime \prime }$) to account for an imbalance in class frequency (3,374 WGD to 1,236 SSD). The data set was split 80–20 for training and testing.

Feature importances were calculated using the rfpimp Python package (Parr and Turgutlu 2020), using permutation importance as the method of calculation. This method was chosen over the default feature importances from sklearn, which are based on mean impurity decrease, as this method can be biased by variable scale or number of categories (Strobl et al. 2007). Permutation importance records the drop in accuracy caused by randomly permuting each feature relative to a base accuracy.

Model training and feature importance calculation was repeated 100 times using the final hyperparameters and feature set with each pass using different randomly selected training and validation data sets in order to estimate how variable the importance rankings were due to randomness in the model.

To control for the effects of duplicate age differences, another model was constructed and importances calculated in the same manner using residuals from regressing each feature on age (equation of the form feature∼age) as input under the assumption these values capture variation in the feature not explained by age variation. Additional models were also constructed using scaled and centered data (Z-scores for each value) in order to check for any effects of differences in scale between features on the importance estimates.

Correlation can impact feature importance estimates, reducing importance for individual features. This issue becomes more prevalent with increasing correlation strength and number of correlated features (Gregorutti et al. 2017), as the impact of removing/permuting a single feature in the group becomes less and less with an increasing number of other correlated features to compensate the lost information. To investigate this, we defined two groups of correlated features (shown in table 2, with each feature in the group considered in isolation in order to obtain an importance estimate independent of any compensation from other correlated features. For example, when considering genomic length we would include only genomic length and drop CDS length, mean intron length, intron count, and intron coverage. The same model was constructed but with all but one feature in a given group dropped and the mean importance and accuracy estimates across 100 iterations calculated in the same manner as above. We take this new importance estimate to represent how informative the feature truly is, including information it may share with other correlated features. “Previous rank” was assigned based on the position in the ranked list of mean importance estimates in the model including all features. “New rank” was assigned based on the position the new mean importance would place the feature under consideration within the previous ranked list. Rank and mean accuracy comparisons are given in table 2 and supplementary tables 2–4, Supplementary Material online for all variations on the data set. Considering these features in isolation seems to indicate that correlation has impacted the importance estimates in our base data analysis. In the base data model, any of genomic length, CDS length, and intron count would be ranked second when considered in isolation while they rank third, fourth, and fifth, respectively when considered together. Additionally, we see very little impact on model accuracy when only considering one feature from the group, suggesting that only one of these features is required to supply almost the same amount of information to the classifier as the entire group. When we consider the age-controlled model, there is comparatively little impact on rankings when features are considered in isolation. Similarly to previous analysis, we do not see any real impact on using scaled data (supplementary table 3, Supplementary table 4, Supplementary Material online).

## Supplementary Material

evad174_Supplementary_Data

## Data Availability

Data used/generated in this work are either publicly available as described in the methods or available at https://github.com/ZoeVance/duplicateComparison

## Supplementary Material

Figures 1 to 11 and Tables 1 to 10 are available at *Genome Biology and Evolution* online.
