# Supplementary material for: Ohnologs and SSD Paralogs Differ in Genomic and Expression Features Related to Dosage Constraints
Source: Genome Biol Evol. 2023 Sep 30;15(10):evad174. doi: 10.1093/gbe/evad174 (PMC10563793; doi:10.1093/gbe/evad174)
Supplement: evad174_Supplementary_Data [file evad174_supplementary_data.pdf]

Ohnologs and SSD paralogs differ in genomic and expression features related to dosage constraints - Supplementary Information

Zoe Vance[1] and Aoife McLysaght[1,2]

[1] *Smurfit Institute of Genetics, Trinity College Dublin, Dublin 2, Ireland*
[2] *Correspondence to aoife.mclysaght@tcd.ie*

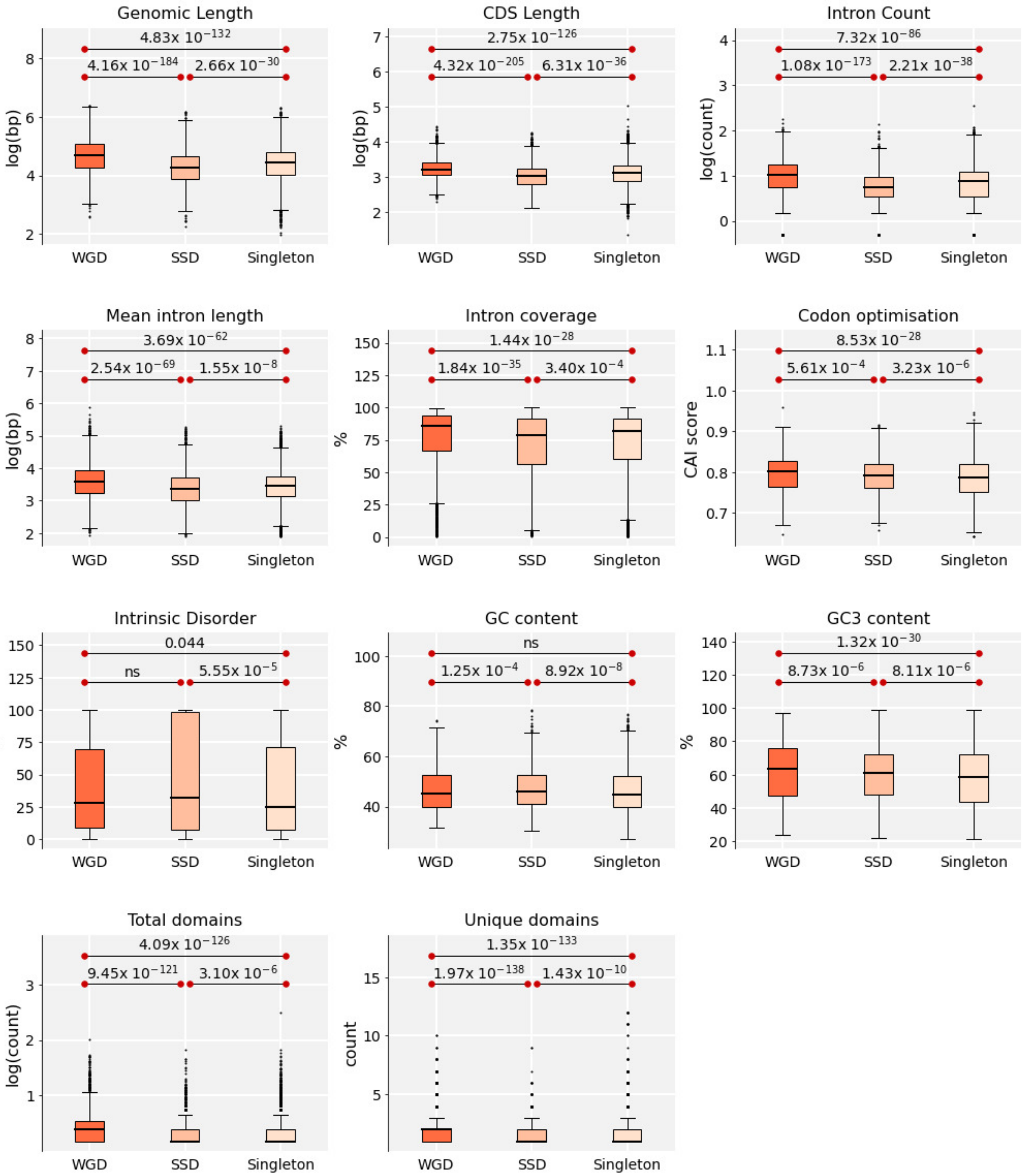

Supplementary Figure 1: All gene structure and sequence based feature comparisons. Comparisons for features classed as related to genomic sequence or gene structure. For each feature, different categories of gene were compared using the Mann-Whitney U test All P-values are Bonferroni-corrected.

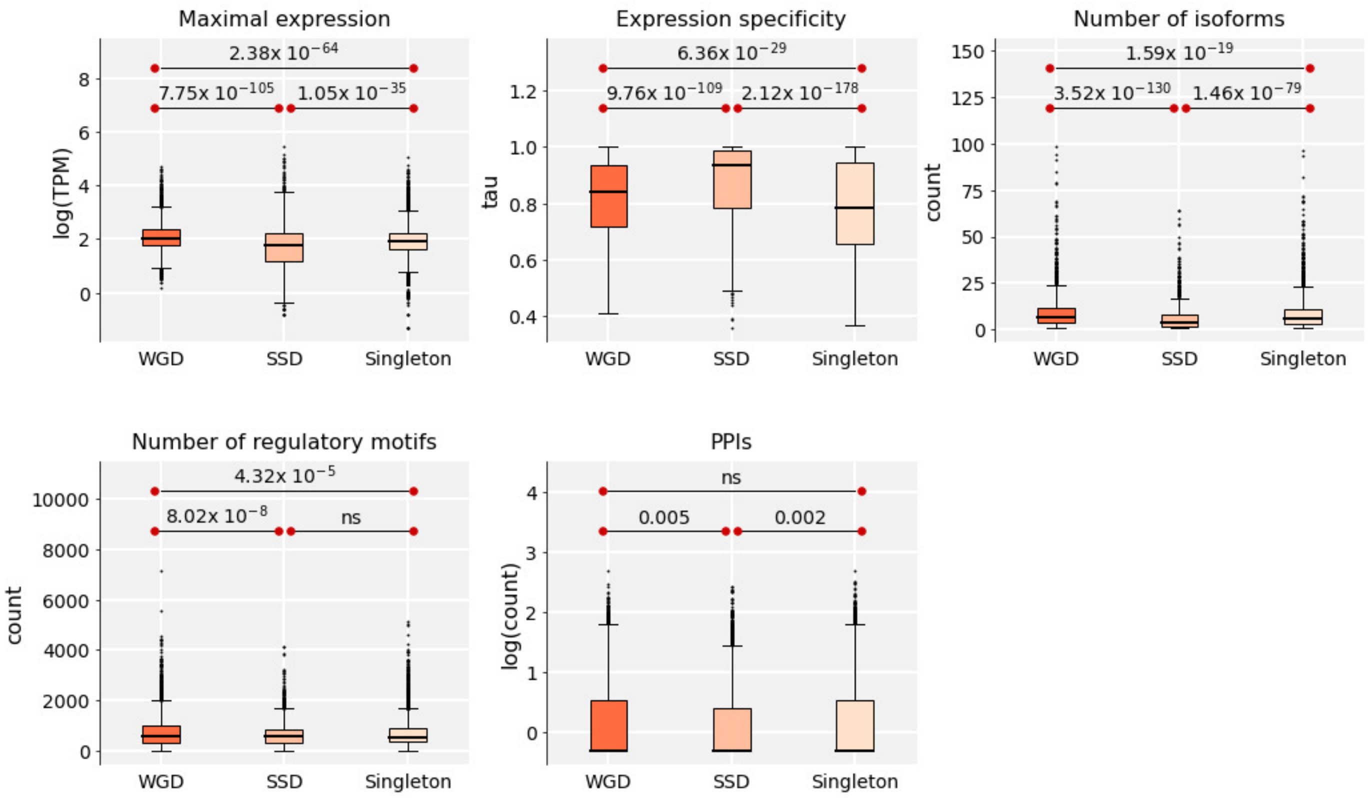

Supplementary Figure 2: All regulation based feature comparisons. Comparisons for features relating to gene features affecting gene expression or behaviour carried out using the Mann-Whitney U Test. All $P-$values are Bonferroni-corrected.

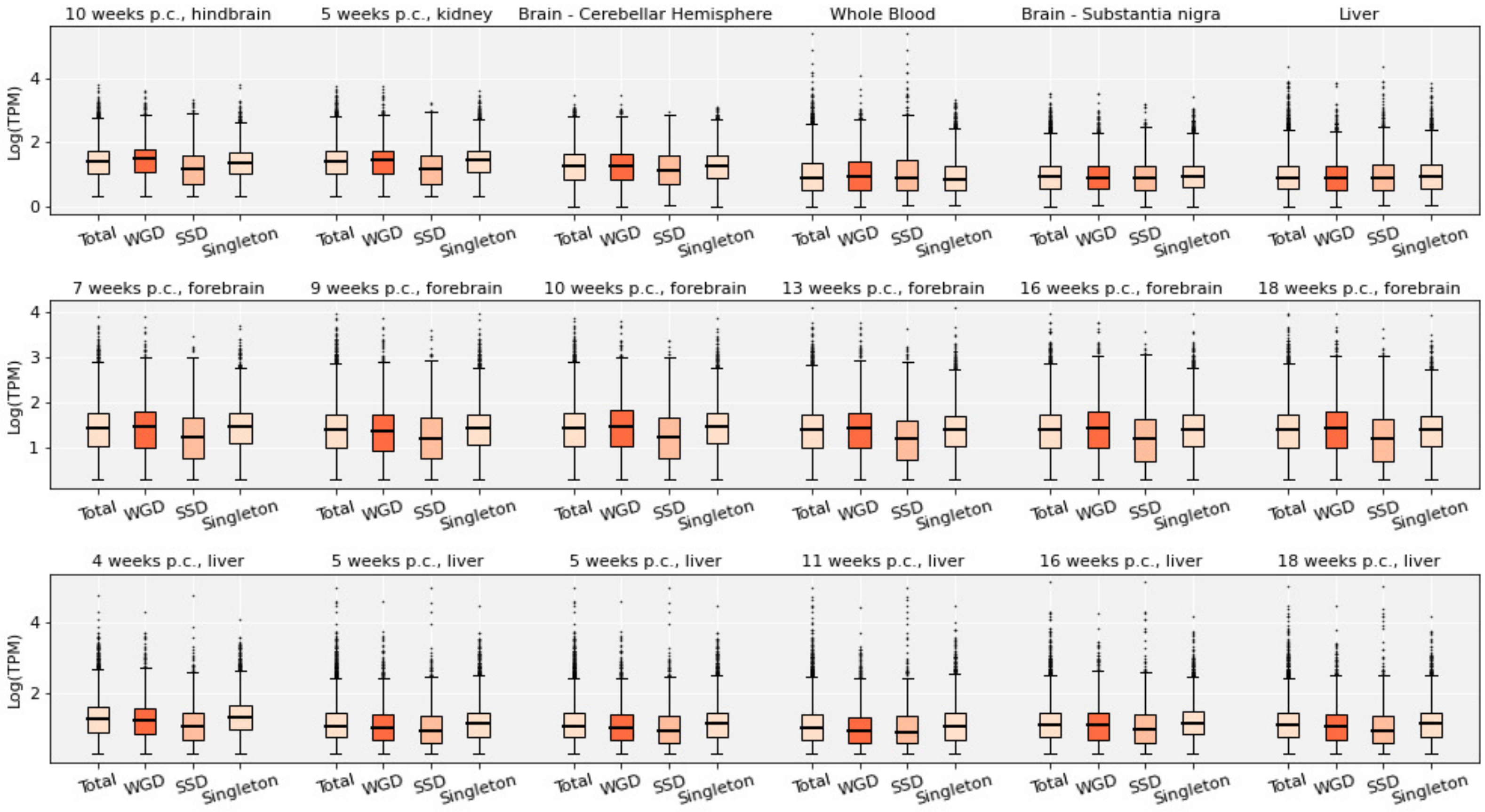

Supplementary Figure 3: Comparison of expression at the tissue level. The top panel shows representative tissues with the highest and lowest difference in median expression between ohnologs and SSDs. The lower two panels show change over development time for a tissue that shows large differences (forebrain) and one which shows a comparatively small difference (liver). 'Total' refers to the distribution of all genes in the genome.

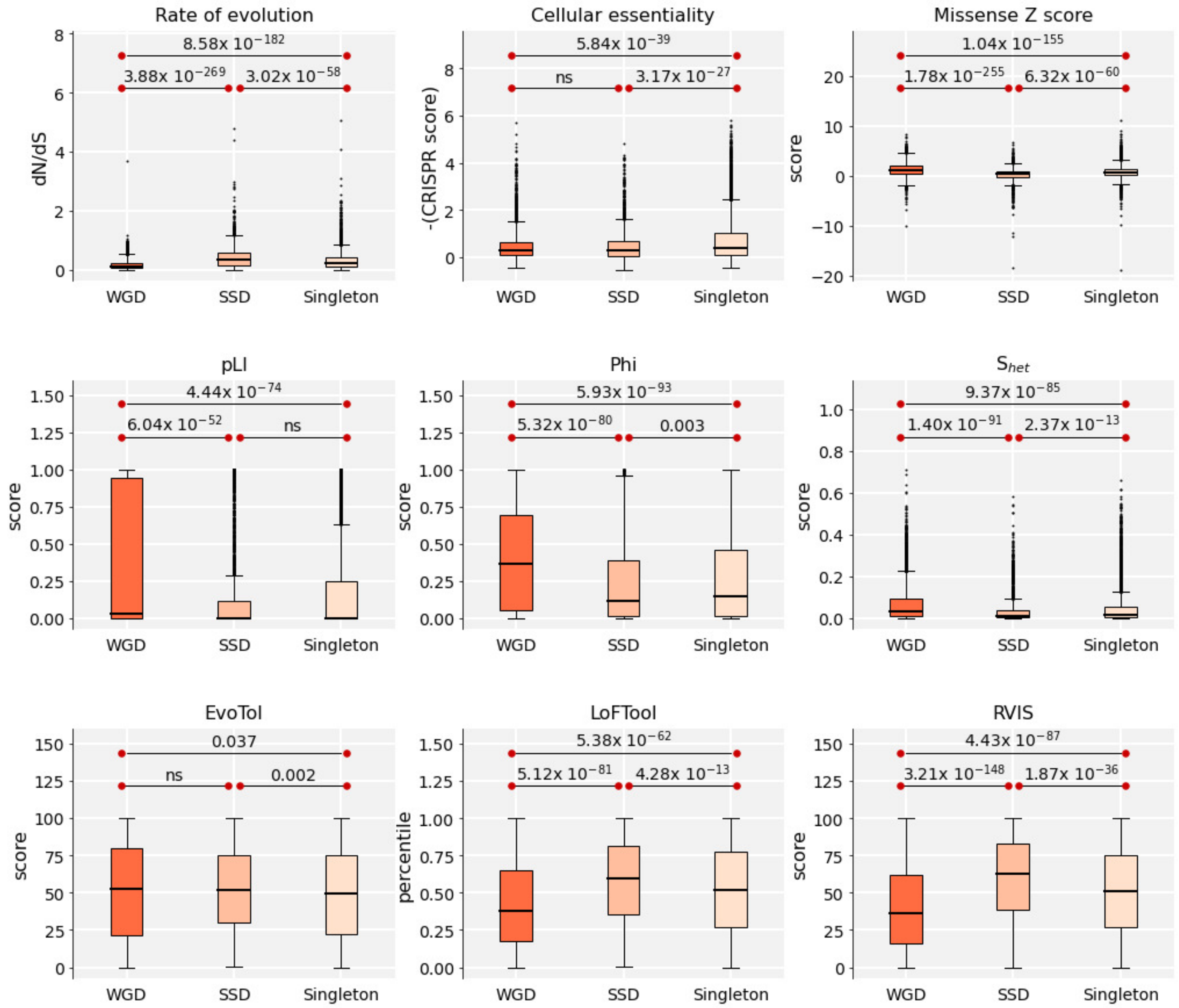

Supplementary Figure 4: All constraint based feature comparisons. Comparisons for features relating to how constrained or essential a gene is. Rate of evolution given by dn/dS, cellular essentiality by CRISPR score (Wang et al. 2015), missense Z score by the Z-score of missense variation in a gene compared to a null model. pLI = probability of LOF intolerance, Phi = probability of haploinsufficiency, S_{het} = selective effects on heterozygous protein truncating variants, EvoTol = evolutionary tolerance, LOFtool = percentile for LOFtool score (lower percentiles are more intolerant to variation), RVIS = residual variance intolerance score. For each feature, different categories of gene were compared using the Mann-Whitney U test. All P-values are Bonferroni-corrected.

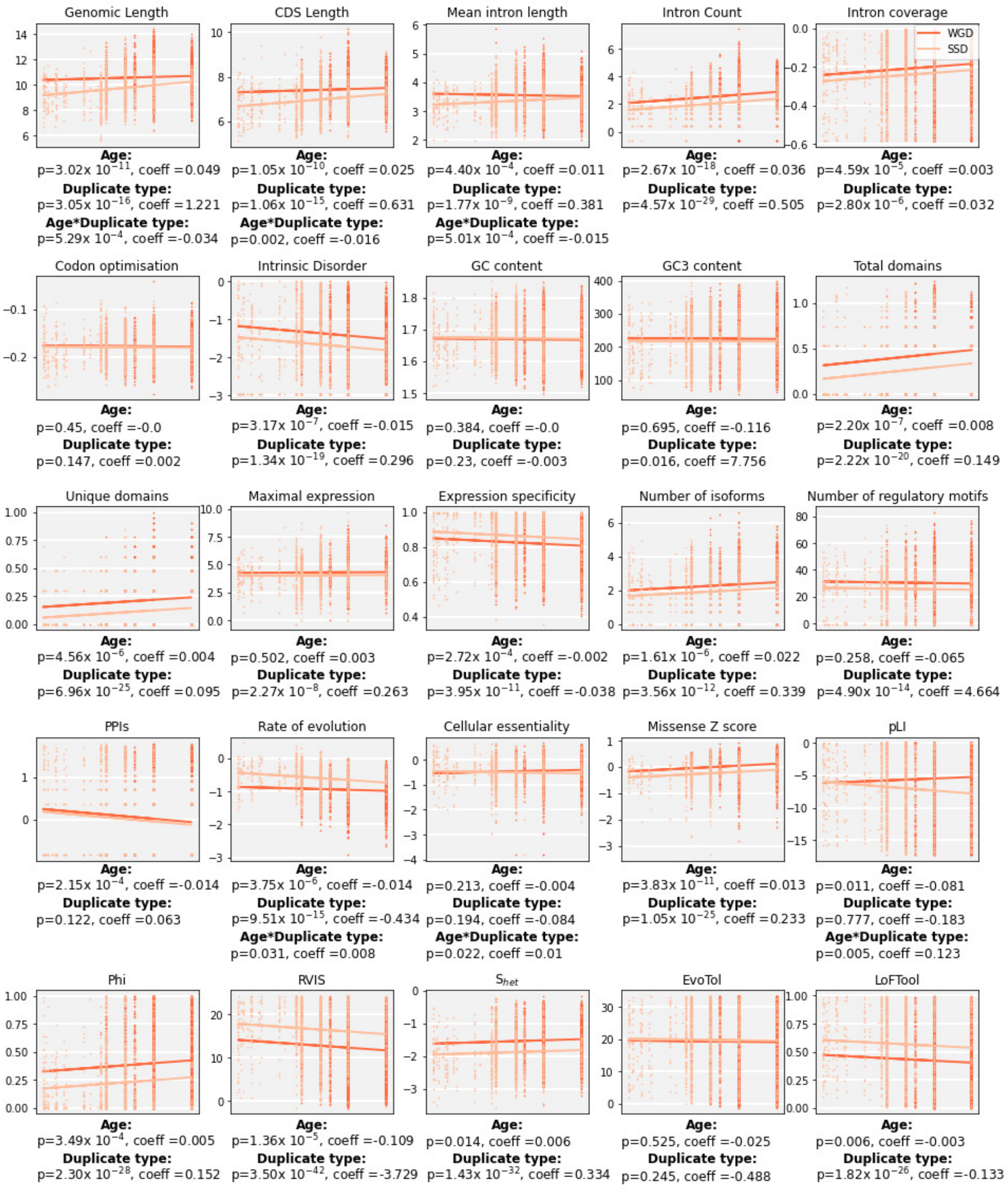

Supplementary Figure 5: Features regressed on duplicate age and duplicate type. Scatter and regression line for each feature with age increasing left to right. WGD shown in darker colour, SSD in lighter. Co-efficients and associated p values for each predictor are given, including for the interaction term where applicable (see Methods). Units for the features are as in previous figures but transformed according to formulas given in Supplementary Table 1
.

Supplementary Table 1: Regression formulae. Transformations for each feature and predictors included in the regression model.

Feature	Regression formula
Genomic Length	$\mathit{Box\text{-}Cox(Genomic\ Length)} \sim \mathit{Age} + \mathit{Duplication\_type} + \mathit{Age} * \mathit{Duplication\_type}$
CDS Length	$\mathit{Box\text{-}Cox(CDS\ Length)} \sim \mathit{Age} + \mathit{Duplication\_type} + \mathit{Age} * \mathit{Duplication\_type}$
Rate of evolution	$\mathit{Log(Rate\ of\ evolution)} \sim \mathit{Age} + \mathit{Duplication\_type} + \mathit{Age} * \mathit{Duplication\_type}$
Unique domains	$\mathit{Log(Unique\ domains)} \sim \mathit{Age} + \mathit{Duplication\_type}$
GC content	$\mathit{Log(GC\ content)} \sim \mathit{Age} + \mathit{Duplication\_type}$
GC3 content	$\mathit{Box\text{-}Cox(GC3\ content)} \sim \mathit{Age} + \mathit{Duplication\_type}$
Total domains	$\mathit{Box\text{-}Cox(Total\ domains)} \sim \mathit{Age} + \mathit{Duplication\_type}$
Expression specificity	$\mathit{Expression\ specificity} \sim \mathit{Age} + \mathit{Duplication\_type}$
Number of isoforms	$\mathit{Box\text{-}Cox(Number\ of\ isoforms)} \sim \mathit{Age} + \mathit{Duplication\_type}$
Intron Count	$\mathit{Box\text{-}Cox(Intron\ Count)} \sim \mathit{Age} + \mathit{Duplication\_type}$
Mean intron length	$\mathit{Log(Mean\ intron\ length)} \sim \mathit{Age} + \mathit{Duplication\_type} + \mathit{Age} * \mathit{Duplication\_type}$
Maximal expression	$\mathit{Box\text{-}Cox(Maximal\ expression)} \sim \mathit{Age} + \mathit{Duplication\_type}$
Cellular essentiality	$\mathit{Log(Cellular\ essentiality)} \sim \mathit{Age} + \mathit{Duplication\_type} + \mathit{Age} * \mathit{Duplication\_type}$
Number of regulatory motifs	$\mathit{Box\text{-}Cox(Number\ of\ regulatory\ motifs)} \sim \mathit{Age} + \mathit{Duplication\_type}$
PPIs	$\mathit{Box\text{-}Cox(PPIs)} \sim \mathit{Age} + \mathit{Duplication\_type}$
Intron coverage	$\mathit{Box\text{-}Cox(Intron\ coverage)} \sim \mathit{Age} + \mathit{Duplication\_type}$
Missense Z score	$\mathit{Log(Missense\ Z\ score)} \sim \mathit{Age} + \mathit{Duplication\_type}$
pLI	$\mathit{Box\text{-}Cox(pLI)} \sim \mathit{Age} + \mathit{Duplication\_type} + \mathit{Age} * \mathit{Duplication\_type}$
LoFTool	$\mathit{LoFTool} \sim \mathit{Age} + \mathit{Duplication\_type}$
S_{het}	$\mathit{Log}(S_{het}) \sim \mathit{Age} + \mathit{Duplication\_type}$
Phi	$\mathit{Phi} \sim \mathit{Age} + \mathit{Duplication\_type}$
RVIS	$\mathit{Box\text{-}Cox(RVIS)} \sim \mathit{Age} + \mathit{Duplication\_type}$
EvoTol	$\mathit{Box\text{-}Cox(EvoTol)} \sim \mathit{Age} + \mathit{Duplication\_type}$
Codon optimisation	$\mathit{Box\text{-}Cox(Codon\ optimisation)} \sim \mathit{Age} + \mathit{Duplication\_type}$
Intrinsic Disorder	$\mathit{Box\text{-}Cox(Intrinsic\ Disorder)} \sim \mathit{Age} + \mathit{Duplication\_type}$

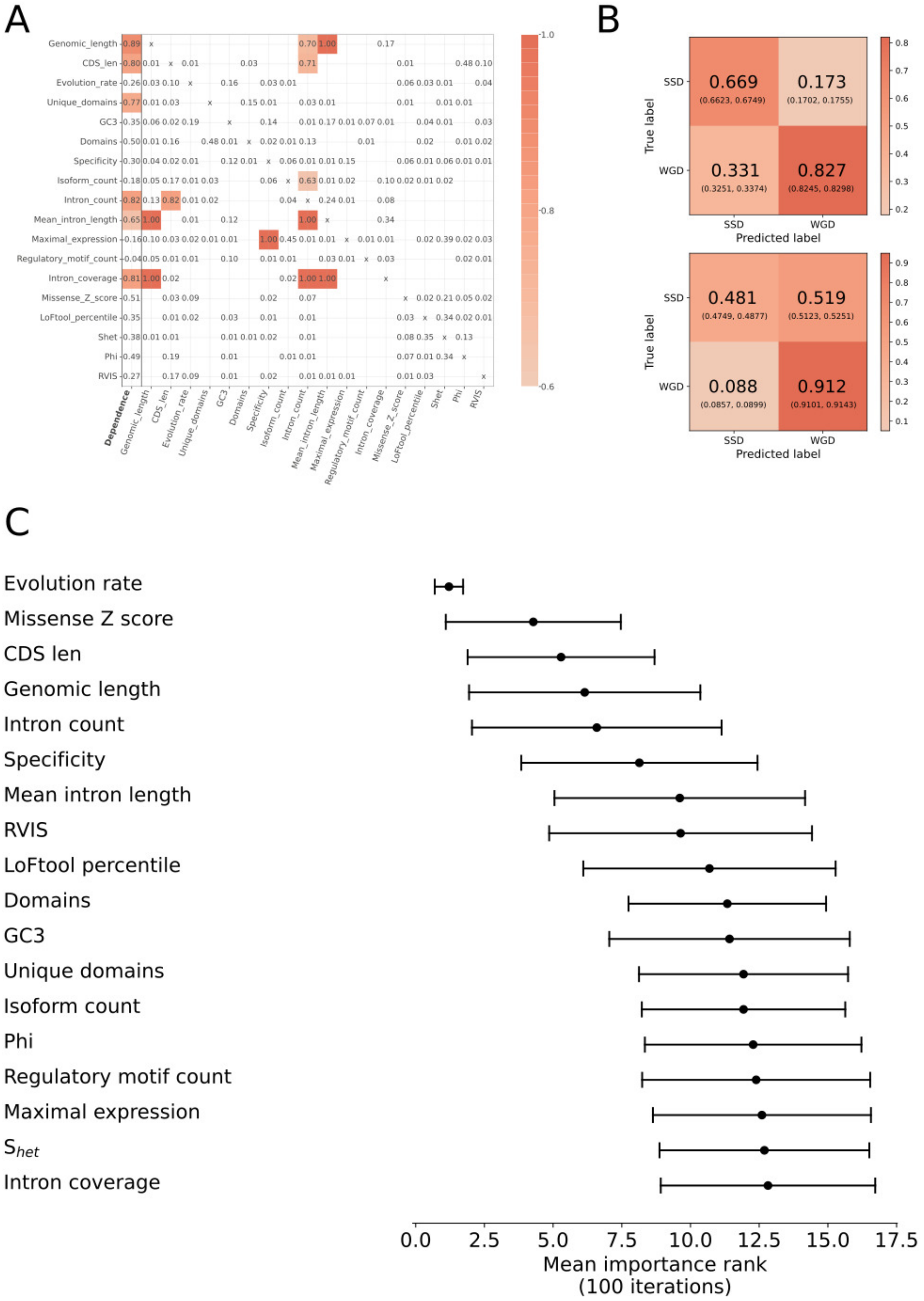
A
B
C
Genomic_length
CDS_len
Evolution_rate
Unique_domains
GC3
Domains
Specificity
Isoform_count
Intron_count
Mean_intron_length
Maximal_expression
Regulatory_motif_count
Intron_coverage
Missense_Z_score
LoFtool_percentile
Shet
Phi
RVIS
Dependence
True label
Predicted label
SSD
WGD
0.669
(0.6623, 0.6749)
0.173
(0.1702, 0.1755)
0.331
(0.3251, 0.3374)
0.827
(0.8245, 0.8298)
0.481
(0.4749, 0.4877)
0.519
(0.5123, 0.5251)
0.088
(0.0857, 0.0899)
0.912
(0.9101, 0.9143)
Evolution rate
Missense Z score
CDS len
Genomic length
Intron count
Specificity
Mean intron length
RVIS
LoFtool percentile
Domains
GC3
Unique domains
Isoform count
Phi
Regulatory motif count
Maximal expression
S_{het}
Intron coverage
0.0
2.5
5.0
7.5
10.0
12.5
15.0
17.5
Mean importance rank
(100 iterations)

Supplementary Figure 6: Duplicate type prediction. (A) Feature dependencies, based on a fitted classifier with each feature as the target and all other features as predictors. The 'Dependence' column gives the R^2 value for the model i.e. how well the feature can be predicted by the others. Other values give the importance of each variable in the given predictive model. Values are bounded at 0 and 1, zero values not shown. (B) Normalised confusion matrix for the random forest classifier, values normalised to 1 within each column. (C) Average importance rank for each feature tested across 100 iterations of fitting the classifier, features ordered by mean importance. Error bars indicate 1 s.d.

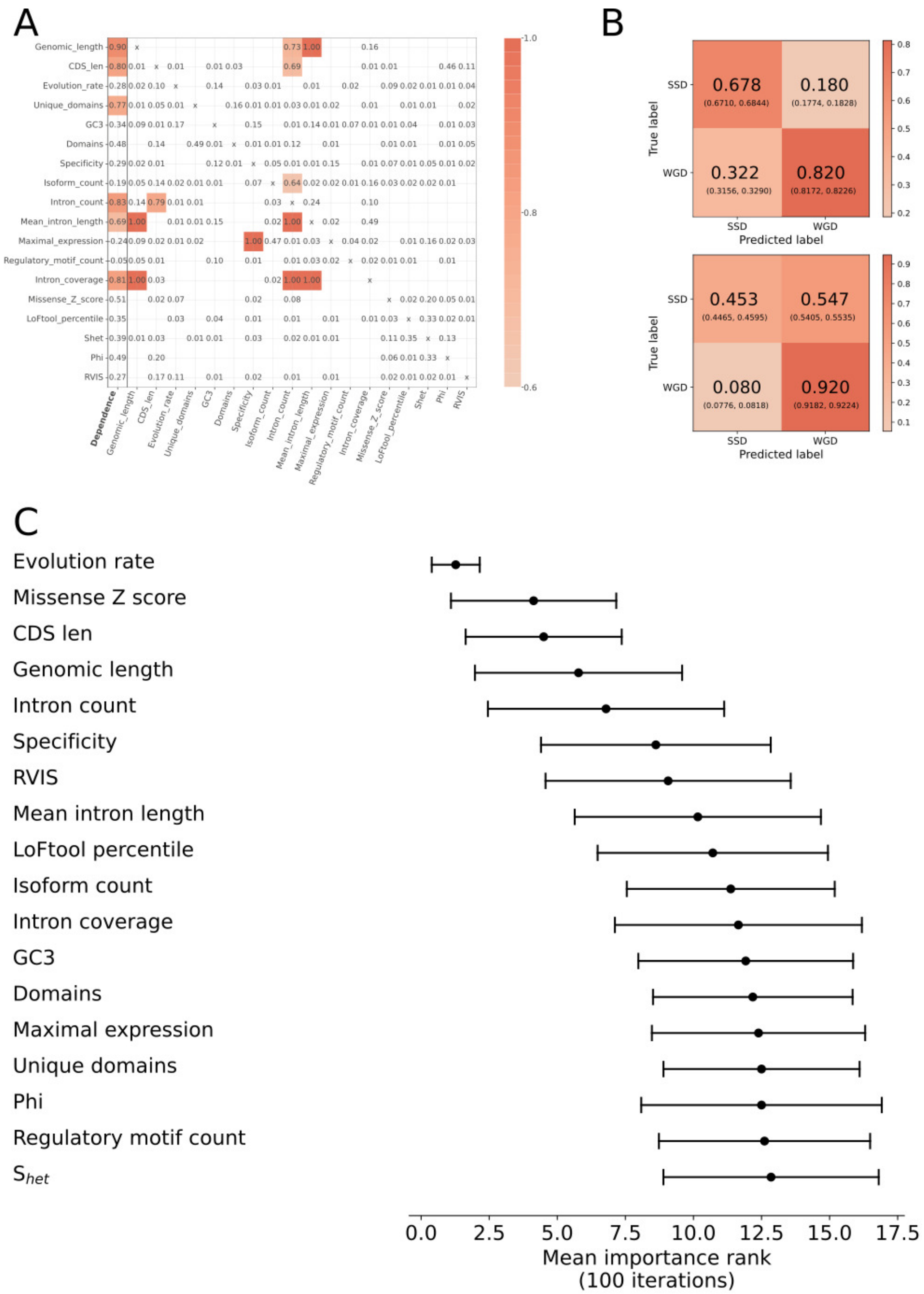
A
Genomic_length
CDS_len
Evolution_rate
Unique_domains
GC3
Domains
Specificity
Isoform_count
Intron_count
Mean_intron_length
Maximal_expression
Regulatory_motif_count
Intron_coverage
Missense_Z_score
LoFtool_percentile
Shet
Phi
RVIS
Dependence
B
SSD
WGD
True label
Predicted label
0.678
(0.6710, 0.6844)
0.180
(0.1774, 0.1828)
0.322
(0.3156, 0.3290)
0.820
(0.8172, 0.8226)
0.453
(0.4465, 0.4595)
0.547
(0.5405, 0.5535)
0.080
(0.0776, 0.0818)
0.920
(0.9182, 0.9224)
C
Evolution rate
Missense Z score
CDS len
Genomic length
Intron count
Specificity
RVIS
Mean intron length
LoFtool percentile
Isoform count
Intron coverage
GC3
Domains
Maximal expression
Unique domains
Phi
Regulatory motif count
S_het
0.0
2.5
5.0
7.5
10.0
12.5
15.0
17.5
Mean importance rank
(100 iterations)

Supplementary Figure 7: Duplicate type prediction using scaled data. (A) Feature dependencies, based on a fitted classifier with each feature as the target and all other features as predictors. The 'Dependence' column gives the R^2 value for the model i.e. how well the feature can be predicted by the others. Other values give the importance of each variable in the given predictive model. Values are bounded at 0 and 1, zero values not shown. (B) Normalised confusion matrix for the random forest classifier, values normalised to 1 within each column. (C) Average importance rank for each feature tested across 100 iterations of fitting the classifier, features ordered by mean importance. Error bars indicate 1 s.d.

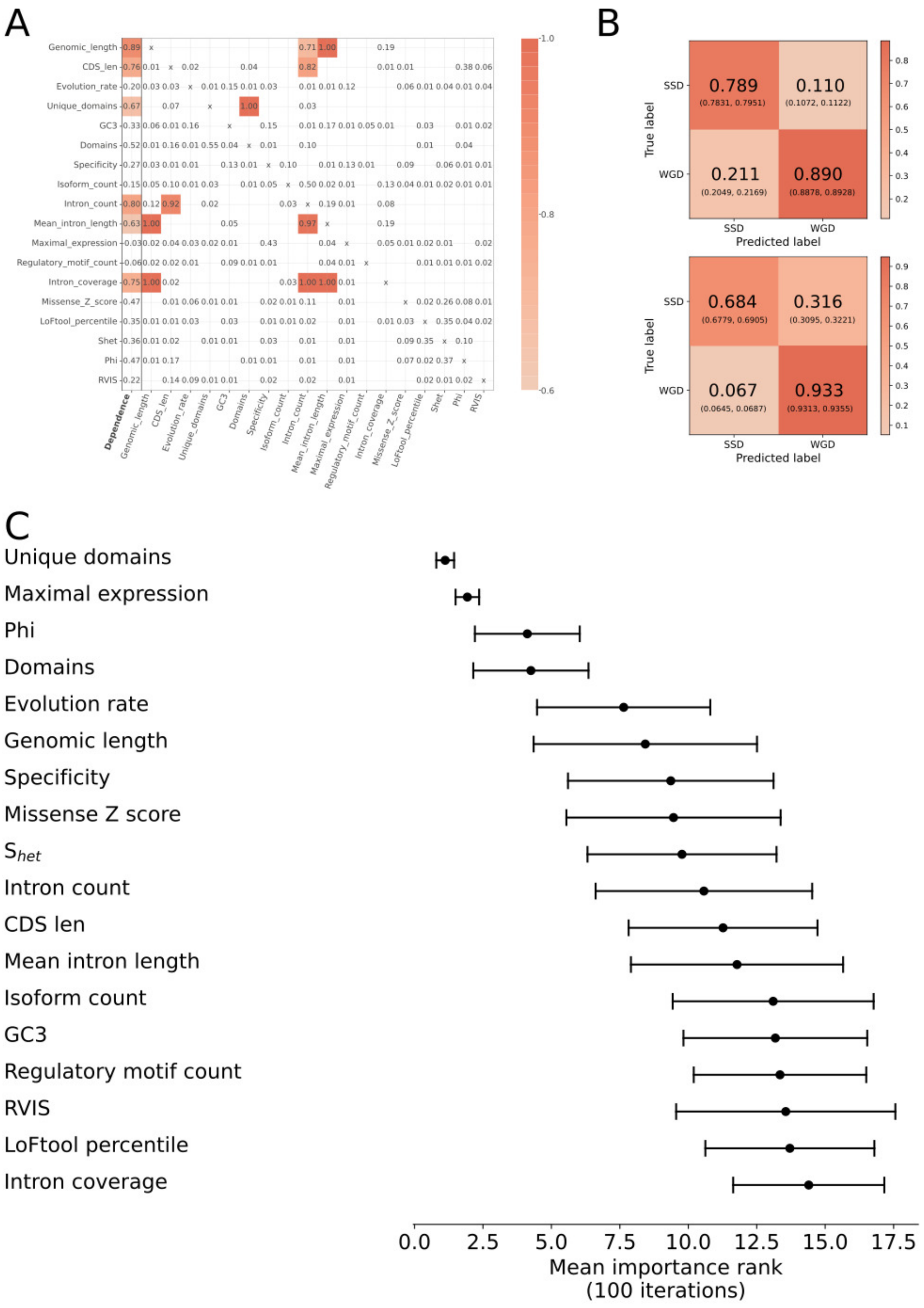
A
Genomic_length
CDS_len
Evolution_rate
Unique_domains
GC3
Domains
Specificity
Isoform_count
Intron_count
Mean_intron_length
Maximal_expression
Regulatory_motif_count
Intron_coverage
Missense_Z_score
LoFtool_percentile
Shet
Phi
RVIS
Dependence
B
SSD
WGD
True label
Predicted label
0.789
(0.7831, 0.7951)
0.110
(0.1072, 0.1122)
0.211
(0.2049, 0.2169)
0.890
(0.8878, 0.8928)
0.684
(0.6779, 0.6905)
0.316
(0.3095, 0.3221)
0.067
(0.0645, 0.0687)
0.933
(0.9313, 0.9355)
C
Unique domains
Maximal expression
Phi
Domains
Evolution rate
Genomic length
Specificity
Missense Z score
S_{het}
Intron count
CDS len
Mean intron length
Isoform count
GC3
Regulatory motif count
RVIS
LoFtool percentile
Intron coverage
0.0
2.5
5.0
7.5
10.0
12.5
15.0
17.5
Mean importance rank
(100 iterations)

Supplementary Figure 8: Duplicate type prediction with scaled data, controlling for age differences. (A) Feature dependencies, based on a fitted classifier with each feature as the target and all other features as predictors. The 'Dependence' column gives the R^2 value for the model i.e. how well the feature can be predicted by the others. Other values give the importance of each variable in the given predictive model. Values are bounded at 0 and 1, zero values not shown. (B) Normalised confusion matrix for the random forest classifier, values normalised to 1 within each column. (C) Average importance rank for each feature tested across 100 iterations of fitting the classifier, features ordered by mean importance. Error bars indicate 1 s.d.

Supplementary Table 2: Change in rank for correlated features considered in isolation.

Feature	Previous rank	New rank	Previous accuracy	New accuracy
Group 1				
Genomic length	4	2	0.7935	0.7859
CDS length	3	2	0.7935	0.7864
Mean intron length	8	4	0.7935	0.7847
Intron count	5	2	0.7935	0.7821
Intron coverage	15	6	0.7935	0.7807
Group 2				
Unique domains	11	16	0.7935	0.7924
Domains	16	11	0.7935	0.7929

Supplementary Table 3: Change in rank for correlated features considered in isolation using centered data.

Feature	Previous rank	New rank	Previous accuracy	New accuracy
Group 1				
Genomic length	4	2	0.7983	0.7906
CDS length	3	2	0.7983	0.7904
Mean intron length	7	3	0.7983	0.7917
Intron count	5	2	0.7983	0.7882
Intron coverage	18	6	0.7983	0.7850
Group 2				
Unique domains	10	9	0.7983	0.7991
Domains	12	10	0.7983	0.7960

Supplementary Table 4: Change in rank for correlated features considered in isolation for centered data using age controlled model.

Feature	Previous rank	New rank	Previous accuracy	New accuracy
Group 1				
Genomic length	6	6	0.8656	0.8647
CDS length	11	11	0.8656	0.8649
Mean intron length	12	11	0.8656	0.8639
Intron count	9	9	0.8656	0.8640
Intron coverage	17	16	0.8656	0.8632
Group 2				
Unique domains	1	1	0.8656	0.8619
Domains	3	3	0.8656	0.8549

Supplementary Table 5: Top enriched terms in WGD genes by p-value.

GO term	Description	p-value
GO:0048731	system development	1.05^{-138}
GO:0065007	biological regulation	8.42^{-138}
GO:0050794	regulation of cellular process	8.84^{-138}
GO:0048856	anatomical structure development	6.79^{-133}
GO:0032501	multicellular organismal process	2.87^{-130}
GO:0032502	developmental process	1.39^{-129}
GO:0009653	anatomical structure morphogenesis	2.90^{-129}
GO:0050789	regulation of biological process	8.16^{-129}
GO:0007275	multicellular organism development	1.57^{-127}
GO:0007399	nervous system development	2.65^{-122}
GO:0023052	signaling	3.04^{-118}
GO:0009987	cellular process	6.17^{-117}
GO:0007154	cell communication	2.83^{-115}
GO:0022008	neurogenesis	4.44^{-107}
GO:0071944	cell periphery	2.45^{-106}
GO:0008150	biological_process	3.14^{-105}
GO:0030154	cell differentiation	3.61^{-105}
GO:0048869	cellular developmental process	5.56^{-104}
GO:0005886	plasma membrane	6.91^{-104}
GO:0030182	neuron differentiation	3.23^{-103}
GO:0048699	generation of neurons	1.43^{-102}
GO:0048468	cell development	6.18^{-101}
GO:0030054	cell junction	1.33^{-99}
GO:0048522	positive regulation of cellular process	4.03^{-99}
GO:0048518	positive regulation of biological process	8.03^{-96}

Supplementary Table 6: Top depleted terms in WGD genes by p-value.

GO term	Description	p-value
GO:0140098	catalytic activity, acting on RNA	8.55^{-26}
GO:0034660	ncRNA metabolic process	6.14^{-25}
GO:0034470	ncRNA processing	1.68^{-24}
GO:0042254	ribosome biogenesis	6.17^{-24}
GO:0022613	ribonucleoprotein complex biogenesis	3.07^{-23}
GO:0016072	rRNA metabolic process	1.01^{-18}
GO:0006364	rRNA processing	2.02^{-18}
GO:0005759	mitochondrial matrix	7.26^{-18}
GO:1990904	ribonucleoprotein complex	4.38^{-16}
GO:0140053	mitochondrial gene expression	2.37^{-15}
GO:0044391	ribosomal subunit	2.40^{-15}
GO:0006281	DNA repair	6.70^{-15}
GO:0006396	RNA processing	1.60^{-14}
GO:0006399	tRNA metabolic process	1.60^{-14}
GO:0009451	RNA modification	8.21^{-14}
GO:0032543	mitochondrial translation	8.44^{-14}
GO:0006259	DNA metabolic process	2.62^{-13}
GO:0003735	structural constituent of ribosome	3.50^{-13}
GO:0008033	tRNA processing	9.64^{-12}
GO:0098798	mitochondrial protein complex	1.11^{-11}
GO:0005743	mitochondrial inner membrane	5.02^{-11}
GO:0033108	mitochondrial respiratory chain complex assembly	7.37^{-11}
GO:0005840	ribosome	8.08^{-11}
GO:0006415	translational termination	3.81^{-10}
GO:0090305	nucleic acid phosphodiester bond hydrolysis	4.53^{-10}

Supplementary Table 7: Top enriched terms in SSD genes by p-value.

GO term	Description	p-value
GO:0048018	receptor ligand activity	7.78^{-28}
GO:0030546	signaling receptor activator activity	7.78^{-28}
GO:0030545	receptor regulator activity	7.78^{-28}
GO:0008009	chemokine activity	2.07^{-22}
GO:0042379	chemokine receptor binding	3.74^{-22}
GO:0006955	immune response	5.41^{-22}
GO:0098542	defense response to other organism	5.41^{-22}
GO:0140375	immune receptor activity	3.19^{-21}
GO:0006952	defense response	8.88^{-21}
GO:0043207	response to external biotic stimulus	7.92^{-20}
GO:0051707	response to other organism	8.99^{-20}
GO:0005126	cytokine receptor binding	1.25^{-18}
GO:0048020	CCR chemokine receptor binding	1.34^{-18}
GO:0009607	response to biotic stimulus	1.49^{-18}
GO:0042742	defense response to bacterium	4.4^{-17}
GO:0019730	antimicrobial humoral response	1.03^{-15}
GO:0070098	chemokine-mediated signaling pathway	3.92^{-15}
GO:0004896	cytokine receptor activity	3.97^{-15}
GO:0045087	innate immune response	7.03^{-15}
GO:0009617	response to bacterium	1.18^{-14}
GO:0005179	hormone activity	1.22^{-14}
GO:0001906	cell killing	1.63^{-14}
GO:0005576	extracellular region	4.95^{-14}
GO:0009897	external side of plasma membrane	6.03^{-14}
GO:0006959	humoral immune response	6.60^{-14}

Supplementary Table 8: Top depleted terms in SSD genes by p-value.

GO term	Description	p-value
GO:0043226	organelle	2.01^{-72}
GO:0005622	intracellular	1.63^{-71}
GO:0043229	intracellular organelle	5.51^{-67}
GO:0003674	molecular_function	6.62^{-66}
GO:1901363	heterocyclic compound binding	1.64^{-63}
GO:0071840	cellular component organization or biogenesis	2.04^{-63}
GO:0097159	organic cyclic compound binding	3.09^{-63}
GO:0043228	non-membrane-bounded organelle	8.99^{-62}
GO:0043232	intracellular non-membrane-bounded organelle	1.35^{-61}
GO:0031981	nuclear lumen	2.12^{-61}
GO:0009987	cellular process	7.77^{-61}
GO:0016043	cellular component organization	4.39^{-58}
GO:0043233	organelle lumen	5.90^{-57}
GO:0070013	intracellular organelle lumen	5.90^{-57}
GO:0031974	membrane-enclosed lumen	5.90^{-57}
GO:0005829	cytosol	6.94^{-57}
GO:0005654	nucleoplasm	8.16^{-54}
GO:0044237	cellular metabolic process	2.31^{-52}
GO:1901265	nucleoside phosphate binding	3.03^{-52}
GO:0000166	nucleotide binding	3.03^{-52}
GO:0043231	intracellular membrane-bounded organelle	2.17^{-50}
GO:0005737	cytoplasm	3.01^{-50}
GO:0017076	purine nucleotide binding	1.16^{-49}
GO:0032553	ribonucleotide binding	1.19^{-49}
GO:0005488	binding	1.19^{-49}

Supplementary Table 9: Top enriched terms in Singleton genes by p-value.

GO term	Description	p-value
GO:0022613	ribonucleoprotein complex biogenesis	6.75^{-44}
GO:0042254	ribosome biogenesis	4.62^{-42}
GO:1990904	ribonucleoprotein complex	1.66^{-41}
GO:0034660	ncRNA metabolic process	2.00^{-39}
GO:0034470	ncRNA processing	1.00^{-38}
GO:0006396	RNA processing	4.33^{-35}
GO:0005759	mitochondrial matrix	2.19^{-32}
GO:0140098	catalytic activity, acting on RNA	2.59^{-32}
GO:0006364	rRNA processing	6.79^{-32}
GO:0003723	RNA binding	4.58^{-31}
GO:0016072	rRNA metabolic process	5.18^{-31}
GO:0043233	organelle lumen	8.69^{-31}
GO:0070013	intracellular organelle lumen	8.69^{-31}
GO:0031974	membrane-enclosed lumen	8.69^{-31}
GO:0006281	DNA repair	9.62^{-31}
GO:0006259	DNA metabolic process	3.63^{-30}
GO:0005739	mitochondrion	2.01^{-27}
GO:1902494	catalytic complex	2.64^{-26}
GO:0005730	nucleolus	1.64^{-25}
GO:0044391	ribosomal subunit	3.08^{-23}
GO:0005654	nucleoplasm	5.39^{-22}
GO:0098798	mitochondrial protein complex	9.99^{-22}
GO:0140053	mitochondrial gene expression	3.48^{-21}
GO:0032543	mitochondrial translation	7.53^{-21}
GO:0031981	nuclear lumen	1.10^{-20}

Supplementary Table 10: Top depleted terms in Singleton genes by p-value.

GO term	Description	p-value
GO:0071944	cell periphery	2.52^{-111}
GO:0005886	plasma membrane	5.62^{-110}
GO:0023052	signaling	1.25^{-93}
GO:0007154	cell communication	2.05^{-90}
GO:0007165	signal transduction	4.67^{-72}
GO:0005887	integral component of plasma membrane	3.63^{-63}
GO:0031226	intrinsic component of plasma membrane	3.63^{-63}
GO:0032501	multicellular organismal process	1.49^{-61}
GO:0030054	cell junction	3.58^{-61}
GO:0065007	biological regulation	5.61^{-58}
GO:0050794	regulation of cellular process	1.16^{-54}
GO:0048731	system development	3.93^{-53}
GO:0050789	regulation of biological process	1.31^{-52}
GO:0050896	response to stimulus	1.45^{-52}
GO:0045202	synapse	2.89^{-49}
GO:0048856	anatomical structure development	3.95^{-49}
GO:0009653	anatomical structure morphogenesis	6.42^{-49}
GO:0032502	developmental process	6.10^{-48}
GO:0007275	multicellular organism development	2.35^{-45}
GO:0051716	cellular response to stimulus	4.21^{-45}
GO:0007166	cell surface receptor signaling pathway	4.34^{-45}
GO:0040011	locomotion	5.72^{-45}
GO:0016477	cell migration	1.41^{-44}
GO:0003008	system process	1.98^{-44}
GO:0007267	cell-cell signaling	1.04^{-43}

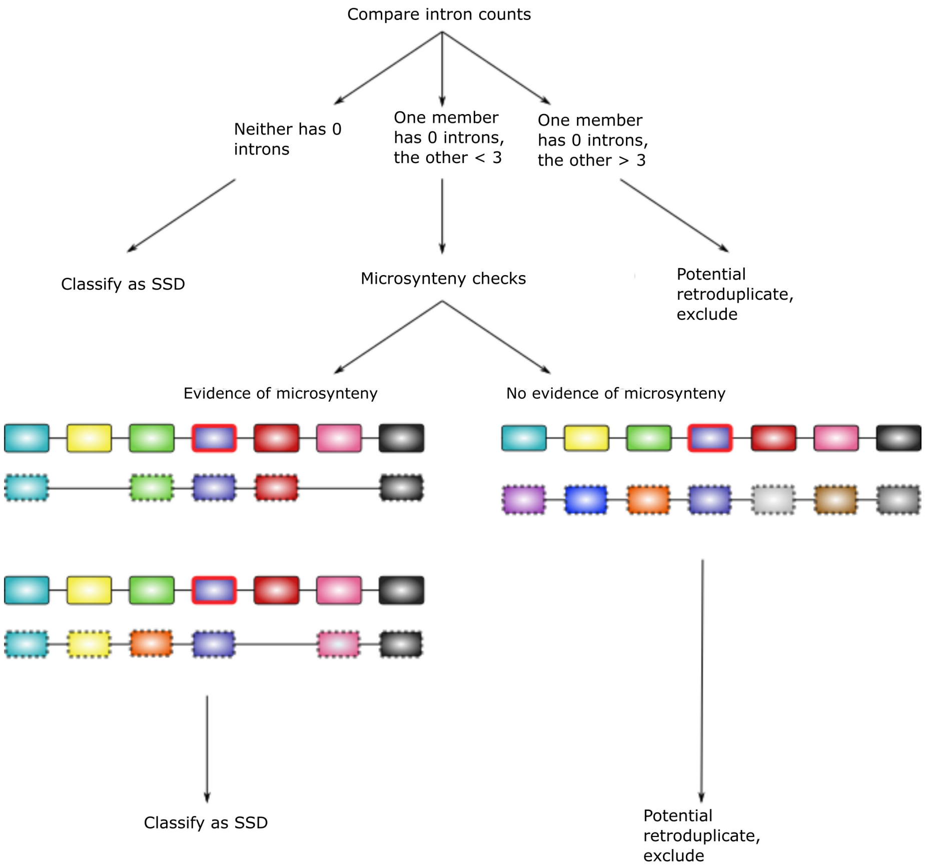

Supplementary Figure 9: Exclusion of retroduplicates. Gene of interest is highlighted by a red outline with neighbouring genes shown on either side. Paralogs of a given gene are shown in the same colour but with a dashed outline. There is no paralogous relationship between genes shown in different colours.

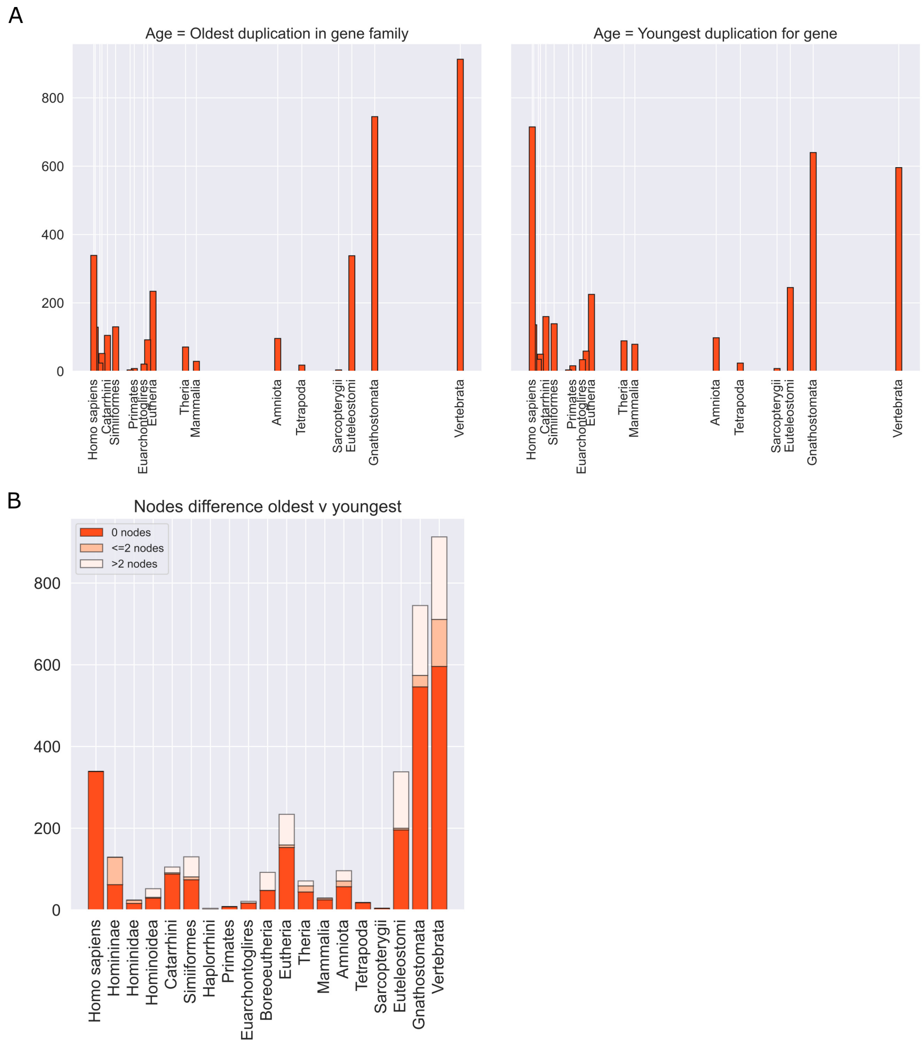
A
Age = Oldest duplication in gene family
Age = Youngest duplication for gene
800
600
400
200
0
Homo sapiens
Catarrhini
Simiiformes
Primates
Euarchontoglires
Eutheria
Theria
Mammalia
Amniota
Tetrapoda
Sarcopterygii
Euteleostomi
Gnathostomata
Vertebrata
B
Nodes difference oldest v youngest
0 nodes
<=2 nodes
>2 nodes
Homo sapiens
Homininae
Hominidae
Hominoidea
Catarrhini
Simiiformes
Haplorrhini
Primates
Euarchontoglires
Boreoeutheria
Eutheria
Theria
Mammalia
Amniota
Tetrapoda
Sarcopterygii
Euteleostomi
Gnathostomata
Vertebrata

Supplementary Figure 10: Impact of using youngest vs. oldest duplication for assigning duplicate longevity. (A) Bar charts showing the number of genes classed as SSDs assigned to each node within the vertebrate phylogeny when considering either the oldest (left) or youngest (right) duplication associated to that gene. Some bars for younger nodes are unlabelled for legibility. (B) Stacked bar chart showing the number of genes with longevity assigned to each node (their oldest duplication) that have their youngest duplication within 0, 2 or more nodes.

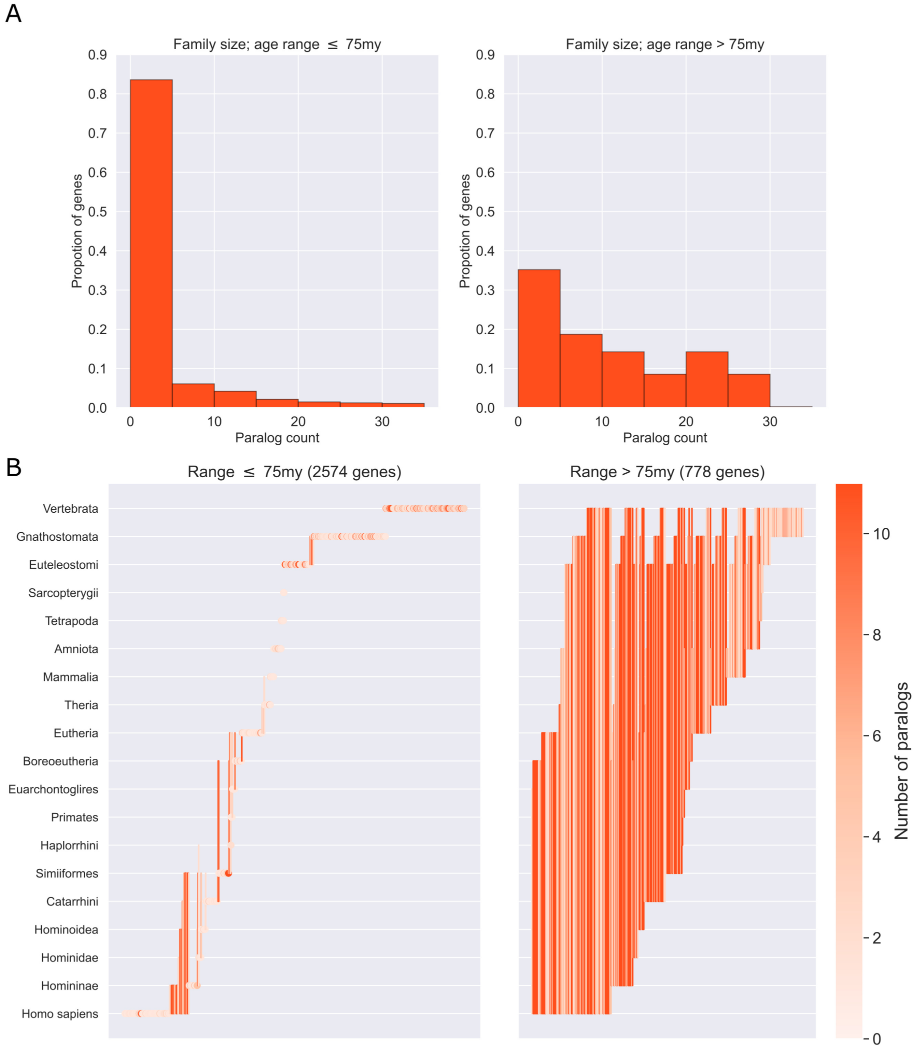
A
Family size; age range ≤ 75my
Family size; age range > 75my
Propotion of genes
Paralog count
B
Range ≤ 75my (2574 genes)
Range > 75my (778 genes)
Vertebrata
Gnathostomata
Euteleostomi
Sarcopterygii
Tetrapoda
Amniota
Mammalia
Theria
Eutheria
Boreoeutheria
Euarchontoglires
Primates
Haplorrhini
Simiiformes
Catarrhini
Hominoidea
Hominidae
Homininae
Homo sapiens
Number of paralogs

Supplementary Figure 11: Genes with a large range in duplication timing come from larger families. (A) Histograms comparing the family size (number of human paralogs) of genes where the range between their oldest and youngest duplication is $\leq$ 75my (left) vs $>$ 75my (right) (B) Range between oldest and youngest duplications for each gene shown as either a line linking the two or a circle if all duplications are dated to the same node. Colour is determined by the number of human paralogs for each gene.

Supplementary Table 11: Default settings for random forest classifier

Parameter	Value
bootstrap	True
ccp_alpha	0.0
class_weight	None
criterion	gini
max_depth	None
max_features	auto
max_leaf_nodes	None
max_samples	None
min_impurity_decrease	0.0
min_impurity_split	None
min_samples_leaf	1
min_samples_split	2
min_weight_fraction_leaf	0.0
n_estimators	100
n_jobs	None
oob_score	False
random_state	None
verbose	0
warm_start	False
